# MWB_Analyzer: An Automated Embedded System for Real-Time Quantitative Analysis of Morphine Withdrawal Behaviors in Rodents

**DOI:** 10.3390/toxics13070586

**Published:** 2025-07-14

**Authors:** Moran Zhang, Qianqian Li, Shunhang Li, Binxian Sun, Zhuli Wu, Jinxuan Liu, Xingchao Geng, Fangyi Chen

**Affiliations:** 1 Department of Biomedical Engineering, Southern University of Science and Technology, Shenzhen 518055, China; 12332581@mail.sustech.edu.cn (M.Z.); 12332584@mail.sustech.edu.cn (S.L.); 12132643@mail.sustech.edu.cn (B.S.); 12331299@mail.sustech.edu.cn (Z.W.); 2 National Center for Safety Evaluation of Drugs (NCSED), National Institutes for Food and Drug Control, Beijing 102629, China; liqianqian@nifdc.org.cn; 3 Shenzhen Giant (Ju’An) Technologies Co., Ltd., Shenzhen 518000, China; aa335263736@gmail.com; 4 Guangdong Provincial Key Laboratory of Advanced Biomaterials, Southern University of Science and Technology, Shenzhen 518055, China

**Keywords:** morphine withdrawal, drug toxicity, automated behavioral analysis, opioid dependence model, YOLO-based video classification, rodent neurotoxicity, high-throughput behavioral screening, addiction pharmacodynamics, CNS-active drug evaluation, preclinical toxicology

## Abstract

**Background/Objectives:** Substance use disorders, particularly opioid addiction, continue to pose a major global health and toxicological challenge. Morphine dependence represents a significant problem in both clinical practice and preclinical research, particularly in modeling the pharmacodynamics of withdrawal. Rodent models remain indispensable for investigating the neurotoxicological effects of chronic opioid exposure and withdrawal. However, conventional behavioral assessments rely on manual observation, limiting objectivity, reproducibility, and scalability—critical constraints in modern drug toxicity evaluation. This study introduces MWB_Analyzer, an automated and high-throughput system designed to quantitatively and objectively assess morphine withdrawal behaviors in rats. The goal is to enhance toxicological assessments of CNS-active substances through robust, scalable behavioral phenotyping. **Methods:** MWB_Analyzer integrates optimized multi-angle video capture, real-time signal processing, and machine learning-driven behavioral classification. An improved YOLO-based architecture was developed for the accurate detection and categorization of withdrawal-associated behaviors in video frames, while a parallel pipeline processed audio signals. The system incorporates behavior-specific duration thresholds to isolate pharmacologically and toxicologically relevant behavioral events. Experimental animals were assigned to high-dose, low-dose, and control groups. Withdrawal was induced and monitored under standardized toxicological protocols. **Results:** MWB_Analyzer achieved over 95% reduction in redundant frame processing, markedly improving computational efficiency. It demonstrated high classification accuracy: >94% for video-based behaviors (93% on edge devices) and >92% for audio-based events. The use of behavioral thresholds enabled sensitive differentiation between dosage groups, revealing clear dose–response relationships and supporting its application in neuropharmacological and neurotoxicological profiling. **Conclusions:** MWB_Analyzer offers a robust, reproducible, and objective platform for the automated evaluation of opioid withdrawal syndromes in rodent models. It enhances throughput, precision, and standardization in addiction research. Importantly, this tool supports toxicological investigations of CNS drug effects, preclinical pharmacokinetic and pharmacodynamic evaluations, drug safety profiling, and regulatory assessment of novel opioid and CNS-active therapeutics.

## 1. Introduction

Opioids remain the most effective pharmacological agents for managing acute and chronic pain, and they continue to dominate the analgesic market [1]. Nevertheless, their widespread use is marred by serious side effects including sedation, respiratory depression, nausea, addiction, and constipation [2]. Globally, more than 26 million individuals suffer from opioid use disorder (OUD) [3], and in the United States alone, over 80,000 deaths from an opioid overdose were reported in 2021 [4]. A hallmark of opioid dependence is the emergence of withdrawal symptoms upon drug cessation [5,6,7,8]. Among opioids, morphine—extensively used in pain management across diverse patient populations [9,10,11]—remains a core model for studying opioid dependence and withdrawal, providing valuable insight into the neurobiological and behavioral dimensions of addiction.

Investigating withdrawal behavior not only provides insights into the neurophysiological changes underlying drug-induced adaptations but also clarifies how disruptions in positive reinforcement contribute to emotional alterations such as anxiety, irritability, and depression [12,13]. Indeed, studies have linked neural adaptations in key brain regions to drug-seeking behaviors during withdrawal [14] and delineated signaling pathways critical for the extinction of drug-associated memories [15,16]. Behavioral models of addiction are critical not only for understanding drug–receptor interactions and pharmacodynamic mechanisms but also for evaluating emerging treatments such as anti-drug vaccines and targeted delivery systems [17,18]. Additionally, pharmaceutical companies are increasingly assessing the abuse liability of candidate drugs early to optimize their risk/benefit profiles and reduce development costs in drug development [19]. Nonclinical studies on drug dependence, including withdrawal studies, provide critical information on the potential for drug dependence in clinical settings. The data obtained from these nonclinical trials can guide clinical research, inform rational drug use, and raise awareness of abuse potential [20].

Drug dependence and withdrawal behaviors impose a significant social and economic burden [21]. The assessment of abuse and dependence liability has been the subject of numerous regulatory documents issued by agencies such as the U.S. Food and Drug Administration (FDA), the China National Medical Products Administration (NMPA), the European Medicines Agency (EMA), and the International Council for Harmonisation (ICH) [19,22]. Assessing potential dependence through withdrawal behavior helps identify the withdrawal symptoms and dependence characteristics of different drugs, guiding regulatory agencies in drug oversight [23]. Research on withdrawal behavior is essential for developing more evidence-based public health policies [24]. By identifying effective interventions, such research can lead to reduced healthcare costs, decreased incidence of substance use disorders, and lower crime rates [25], thereby yielding significant social and economic benefits.

Preclinical rodent models play a foundational role in investigating the biopharmaceutical properties of opioids, including their absorption, distribution, metabolism [26], and excretion (ADME) profiles [27], as well as drug–receptor interactions and neurobehavioral outcomes [28,29]. These models also support the exploration of pharmacogenetic variability and the validation of emerging neuropharmacological targets [17,18,30]. Both the ICH M3 document and the EMA’s 2006 guideline indicate that rodents are the preferred species for assessing abuse liability unless there is a specific reason to use primates [19]. Experimental methods to assess the physical dependence induced by test substances mainly include spontaneous withdrawal, precipitated withdrawal, and substitution experiments. Whether during spontaneous or precipitated withdrawal, animals exhibit a range of symptoms, although not all withdrawal symptoms manifest in every individual, indicating significant inter-animal variability [20]. Additionally, withdrawal studies in preclinical animal models often lead to animal mortality, necessitating the use of large numbers of experimental animals. Research has shown that for the same batch of rat behavioral images, inter-observer agreement on behavior classification is only 0.65 [31].

Historically, early methods for assessing morphine dependence in rats relied on a single behavioral indicator (e.g., jump frequency) [32,33,34,35]. However, single measures fall short of capturing the full spectrum of withdrawal symptoms, often leading to biased assessments [36]. As the field evolved, comprehensive scoring systems such as the Gellert–Holtzman and Maldonado Scales were developed [37,38]. Although these scales have been adapted in various studies [39,40,41,42], manual evaluation remains labor-intensive, subjective, and prone to inter-observer variability [31]. These limitations hinder the scalability, reproducibility, and translational relevance of behavioral data, especially in high-throughput screening or drug formulation optimization contexts.

In recent years, the field of pharmaceutics has witnessed an increasing integration of automation, engineering innovation, and data-driven methodologies to improve the precision and throughput of drug evaluation processes [43,44,45]. This paradigm shift is especially evident in areas such as controlled-release systems, pharmacokinetic and pharmacodynamic modeling, and biomedical device-assisted drug delivery [46,47,48]. However, the behavioral dimension of drug efficacy and safety assessment—particularly in the context of central nervous system (CNS)-acting drugs—remains relatively underserved by such technologies. There is an urgent need for robust, automated platforms that can quantify complex behavioral phenotypes with high fidelity and consistency.

Recent advancements in automated behavior recognition have enabled the detection of specific rat behaviors including wet-dog shakes, scratching, and rearing [49,50,51,52,53]. Yet, systems capable of simultaneously identifying multiple behaviors in a single animal remain limited, often suffering from reduced accuracy when the behavior categories exceed four [31]. In addition to recognition systems based on object detection and tracking, pose estimation-based universal behavior recognition methods have also been widely explored [54], with the rapid development of pose estimation technology [55,56]. Specifically, these approaches involve first performing pose estimation on model organisms (e.g., mice, fruit flies) using deep learning, followed by downstream tasks of behavior recognition [54]. Moreover, the complexity of deep learning-based pose estimation presents disadvantages in terms of throughput and speed [57,58]. Furthermore, Hu et al. proposed a learning-based approach for comprehensive evaluation and quantification of user-defined animal behaviors [59]. This method not only identifies behavior types but also quantifies them. It integrates multi-animal tracking, pattern recognition, and customizable deep learning to comprehensively evaluate behaviors, making the analysis both efficient and accurate. However, the performance of this system in recognizing stationary behaviors in rats has been unsatisfactory. In summary, although multiple devices can be used to recognize different behaviors associated with morphine withdrawal, they are limited by their inability to simultaneously identify multiple behaviors in the same rat. This makes experimental setups with multiple devices highly vulnerable to the effects of individual variability among animals. While universal behavior recognition systems can identify a broader range of behaviors, they often suffer from inadequate accuracy for certain behaviors, as well as issues with low throughput and slow processing speeds. As a result, not all behavioral recognition needs can be fully met by these systems.

To address the challenges of objective, efficient, and scalable analysis of morphine withdrawal behaviors, we developed a novel behavioral recording and analysis system, MWB_Analyzer, with five key innovations:

(1) Multi-angle video and full-scene audio capture via an edge computing platform for comprehensive data acquisition;
(2) Real-time data reduction algorithms that significantly lower processing loads while preserving critical information;
(3) Created and published a dataset of morphine withdrawal behavior video clips in rats for future research;
(4) Enhanced behavioral recognition accuracy and objectivity through an improved YOLO-based framework and signal processing methods;
(5) A more objective scoring protocol was used to score morphine withdrawal behavior to assess the degree of morphine addiction.

Consequently, MWB_Analyzer offers a robust alternative to traditional manual observation methods, enabling more efficient, objective, and scalable assessments of morphine withdrawal behaviors in preclinical models. By delivering real-time, high-resolution analysis of behavioral phenotypes, the system enhances the sensitivity, reproducibility, and translational value of experimental data—factors critical for investigating the neuropharmacological and toxicological effects of opioids and their antagonists. Its integration into addiction research workflows can accelerate the identification of therapeutic targets, support the screening and optimization of neuroactive compounds, and enable precise dose–response modeling essential for evaluating CNS drug safety and efficacy. Furthermore, MWB_Analyzer aligns with the increasing demand for automated, data-driven platforms in toxicology and behavioral neuroscience, where reproducible endpoints are vital for assessing drug-induced neuroadaptations, withdrawal severity, and CNS toxicity profiles. The system also has translational value in pharmacogenetic and pharmacokinetic research, offering a standardized approach to comparing treatment responses across diverse genetic or biological backgrounds. Ultimately, by bridging engineering innovation with neurotoxicological and behavioral pharmacology, MWB_Analyzer supports interdisciplinary research aimed at advancing opioid safety assessments, guiding rational therapeutic use, and addressing the escalating global burden of opioid dependence and withdrawal.

## 2. Materials and Methods

### 2.1. Animals and Drugs

This study utilized male Sprague-Dawley rats (weighing 250 g–300 g, 8 weeks of age), which were purchased from certified suppliers and housed at the Animal Experiment Center of the Institute of Safety Evaluation, National Institutes for Food and Drug Control (National Center for Drug Safety Evaluation and Research), China. Prior to the experiments, all rats were quarantined for 7 days in a controlled environment to ensure their stable health condition. The experiments were conducted under controlled conditions with a temperature of 22 °C ± 1 °C, humidity of 55% ± 5%, and a 12 h light/dark cycle. The rats were provided with free access to standard laboratory chow and water.

A total of 60 rats were used in this study, divided across three experimental stages. In the first stage, 24 rats were used to construct a behavioral dataset under a high-dose morphine administration protocol. All animals in this stage received subcutaneous injections of high-dose morphine sulfate once daily for 7 consecutive days at escalating doses (10, 20, 30, 40, 50, 60, and 70 mg/kg). The dosage and method of drug administration were based on established protocols for morphine dependence and withdrawal models reported in previous studies [60]. In our earlier work, we further refined these methods to successfully establish a morphine dependence and withdrawal model. These data were used to train and validate the behavioral analysis system. In the second stage, 15 rats were used to explore behavioral assessment schemes for morphine addiction. The animals were randomly assigned to two groups: a treatment group (*n* = 12) receiving the same escalating high-dose morphine regimen as above, and a control group (*n* = 3) receiving an equivalent volume of saline. Due to morphine toxicity, 4 rats in the treatment group died during the experiment, resulting in valid behavioral data from 11 treated rats and 3 control rats. These animals were monitored using the custom-built hardware platform, which operated stably during the entire data collection period. Audio and video data were recorded continuously by a single operator. In the third stage, an independent cohort of 21 rats was used to evaluate the performance and sensitivity of the proposed experimental apparatus (MWB_Analyzer) and behavioral assessment protocol. The animals were divided into three groups: a high-dose group (*n* = 7), receiving the escalating morphine regimen as above; a low-dose group (*n* = 10), receiving half the dose of the high-dose group each day (5, 10, 15, 20, 25, 30, and 35 mg/kg); and a control group (*n* = 4), receiving saline injections. Behavioral withdrawal scores were recorded daily and statistically analyzed using one-way ANOVA followed by Tukey’s multiple comparisons test.

Animals were administered twice daily, with a 6 h interval between each injection. After the completion of the injections on day 7, the proposed system was employed to collect behavioral data from the rats as withdrawal symptoms commenced. The designed algorithm was utilized for the automatic recognition and statistical analysis of various behavioral occurrences. Given that the withdrawal responses of the rats would gradually diminish, the first three days were marked as the most pronounced period [60], thus focusing on behavioral data collected at 12 h (day 1), 36 h (day 2), and 60 h (day 3) post-withdrawal. Here, day 1, day 2, and day 3 refer to the first, second, and third days after the completion of drug administration for model induction.

All studies were carried out in accordance with Good Laboratory Practice (GLP) (National Medical Products Administration [NMPA], 2017) and the Preclinical Safety Evaluation of Biotechnology-Derived Pharmaceuticals (ICH S6, 2011). All animal procedures were approved by the Institutional Animal Care and Use Committee (IACUC) of the National Center for the Safety Evaluation of Drugs (NCSED) (No. IACUC-2024-K001).

### 2.2. Hardware Design and System Integration

The MWB_Analyzer is designed as a practical, easy-to-integrate tool for preclinical researchers. It uses small, cost-effective Orange Pi devices (Chengzi Cloud Computing (Shenzhen) Co., Ltd., Shenzhen, China) to simultaneously record video and audio from multiple angles. The system automatically processes this information in real time, requiring no manual setup of hardware or programming by the user. Once installed, the system runs autonomously and fits seamlessly into standard experimental workflows.

**Figure 1:**
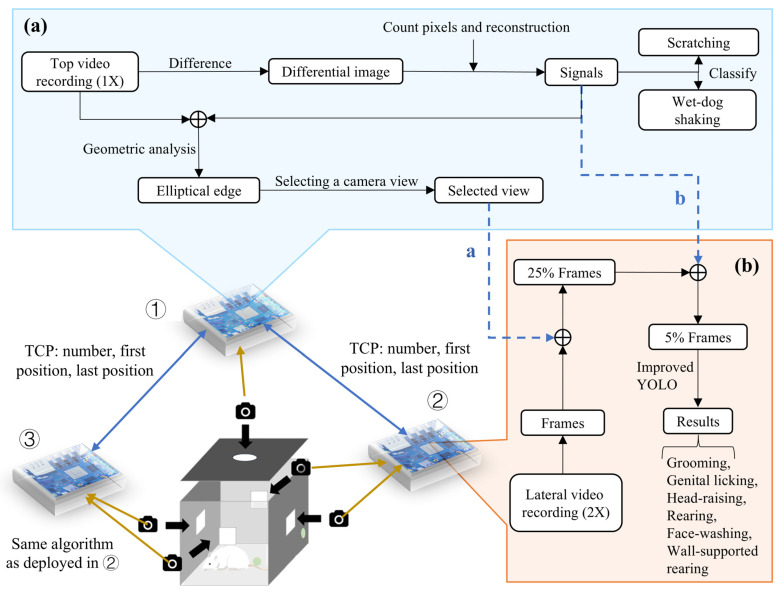
Behavioral video recording and analysis of a single device. (**a**) Video recording and processing workflow of the top Orange Pi (①). (**b**) Video recording and processing workflow of the side Orange Pi (② and ③). a: Communication with either ② or ③ is established based on the calculated viewing angle, transmitting the camera number that needs to be processed via TCP. b: Video clips containing only motion information are filtered from the differential frame signal, and the positions of the first and last frames of the video clips are transmitted to either ② or ③ via TCP.

The design of an acquisition device is illustrated in Figure 1. The structure of the rat cage measures approximately 45 cm × 45 cm × 55 cm, providing enough space for adult rats to move freely and stand upright. Each cage housed a single rat during behavioral recording to avoid social interaction effects. Since rats are unable to perceive infrared light, the system employs infrared light sources, arranged in the form of perforated light panels mounted at the top of the enclosure. This approach not only prevents the potential behavioral impact caused by excessive light intensity but also facilitates the acquisition of high-quality video data. To avoid missing behavioral information due to blind spots in the field of view, the system utilizes a multi-angle design, with USB cameras installed on all four sides and the top of the single-channel enclosure. The cameras used in the system offer excellent data transmission rates and easy control. Additionally, two MEMS digital microphones ensure the high-quality collection of ultrasonic vocalizations.

**Figure 2:**
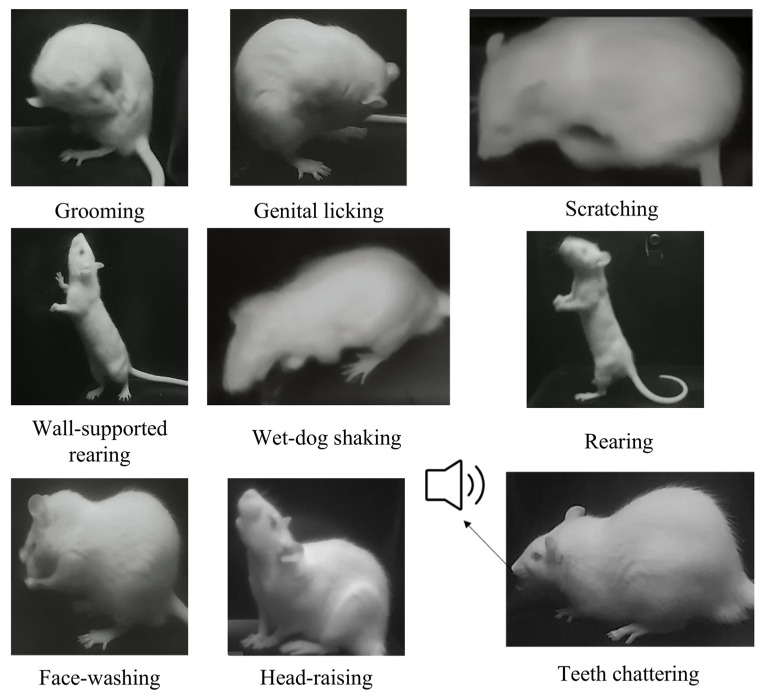
Examples of behaviors measurable by MWB_Analyzer.

Integrated via an Ethernet switch (TP-Link Technologies Co., Ltd., Shenzhen, China) and Orange Pi devices, the system synchronizes video and audio capture using an edge-computing platform (three Orange Pi devices) that processes multi-view data in real time. Examples of behaviors measurable by MWB_Analyzer are illustrated in Figure 2.

### 2.3. Video Capture and External Trigger Synchronization

A multi-threaded video acquisition system built on the Orange Pi device employs five USB cameras and the FFmpeg framework to decode MJPEG streams. Each camera is registered and dynamically configured to ensure proper synchronization through timestamping relative to a common reference. An integrated hardware–software trigger—initiated by a rapid brightness change—minimizes delays and achieves microsecond-level synchronization, which is vital for accurate spatial-temporal analysis.

### 2.4. Motion Segment Filtering and Camera Angle Selection

Using temporal data generated from video frame differencing, we constructed a complete dynamic signal sequence, laying the foundation for subsequent behavioral analysis. In this work, we propose an integrated signal processing pipeline that enhances measurement quality by fusing multi-frame data with adaptive smoothing to amplify motion features and by extracting robust signal segments through envelope detection and baseline correction. This approach reduces data dimensionality and suppresses noise, thereby supporting more accurate behavior analysis. This straightforward process not only significantly reduces the redundancy of raw video data but also ensures the scientific rigor and reliability of behavioral analysis by preserving the key dynamic features. Based on this estimated motion, the system further filters out over 80% of redundant frames, which typically display the rat in a stationary state.

Analyze only the first frame of the video segments filtered from the top view to further reduce the performance overhead. Erosion and dilation processes are applied to remove irrelevant areas such as the tail and other noise. Finally, an elliptical fitting method is used to obtain the rat’s position and orientation. This paper proposes selection criteria based on the rat’s orientation and position to automatically choose the optimal angle from multi-angle videos, improving processing efficiency. This method effectively reduces computational load while ensuring accurate behavior capture and recognition.

We package the camera IDs (corresponding to the best angles), the first frame timestamp, and the last frame timestamp of the remaining 20% of the segments together, and transmit them via TCP to the two side Orange Pi devices. The side Orange Pi devices determine which of the four side video streams should be processed by identifying the camera ID. This means that the side devices only need to process 25% of the video data. Furthermore, the side Orange Pi devices crop the video segments containing motion information based on the first and last frame timestamps, which helps filter out most of the video segments without motion information.

In total, through frame differencing processing, the system efficiently identifies relevant behavioral data, reducing redundant information by over 80%. Multi-angle video recordings enhance the capture of comprehensive behavioral patterns, with specialized top-view camera analysis reducing subsequent processing by 75%. Extensive experiments have shown that only about 5% of the remaining video frames need to be used for recognition, significantly reducing the computational load and improving video processing efficiency.

### 2.5. Wet-Dog Shakes and Scratching Behavior Recognition

Frame-difference analysis calculates the grayscale differences between consecutive frames and applies threshold filtering, retaining only pixels with significant movement, which significantly reduces the amount of data to be processed. This method operates efficiently without requiring high computational power and can accelerate subsequent analyses through parallel processing. Wet-dog shakes and scratching behaviors exhibit distinct short-term rhythmic motions when observed from a top-down perspective using image signals. This characteristic is reflected in the periodic waveform of the signal reconstructed from frame-difference pixel changes. By processing this signal, we can accurately and quickly recognize these behaviors.

To enhance recognition efficiency, we designed a fast identification method based on one-dimensional signal processing, consisting of two steps: an initial screening to eliminate irrelevant signal segments and a fine-tuned process to confirm the final result. The initial screening uses smoothing and peak detection techniques to locate candidate signal segments, while the secondary screening applies stricter multi-peak characteristics to determine the accuracy of the shake signal. This method effectively improves the speed and precision of wet-dog shake behavior identification in video data.

For scratching behavior recognition, the repeated motion of the rat’s paws causes the frame-difference image to show regular and sharp or smooth variations, with the signal manifesting as continuous peaks of a similar height. By extracting multiple time-domain and frequency-domain features and designing appropriate thresholds, scratching behavior can be effectively identified.

### 2.6. Lateral Behavior Recognition

After dimensionality reduction and processing of the data from the top camera, we successfully extracted video segments containing motion information. Through efficient communication between development boards, this information was transmitted to the side board, enabling rapid processing of the corresponding video segments. YOLO (You Only Look Once), an advanced deep learning method for object detection, has achieved significant results across various fields, including industry, medicine, and agriculture.

Behavioral video clips are typically composed of a series of consecutive frames. Even for the same behavior category, frame-to-frame differences can be significant. If the video clips are directly decoded for training and classification, the resulting model may become highly unstable, leading to a decrease in classification performance. In fact, certain prior knowledge can be of considerable help. Specifically, the behaviors of interest often exhibit characteristic events, which correspond to key frames in the video (i.e., frames that capture significant moments). Key frames are typically associated with stable events. Therefore, we can perform classification tasks based on the key frames of behavioral video clips.

**Figure 3:**
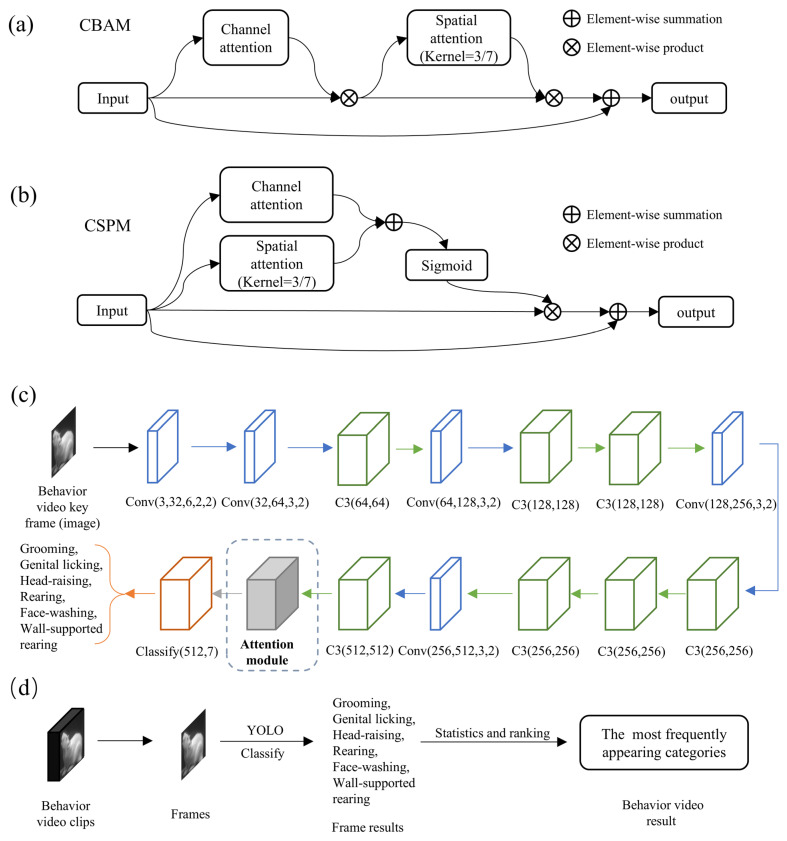
Improved YOLO-based side-view video classification method. (**a**) Basic structure of the CBAM module. (**b**) Basic structure of the CSPM module. (**c**) Basic structure of YOLOv5 image classification and the position of improvements. (**d**) Workflow for video clip classification based on the improved YOLO model.

Specifically, MWB_Analyzer first classifies key frames of the video using the improved YOLO network. Here, we leverage the CBAM module (Figure 3a) from existing research and the self-developed CSPM module (Figure 3b) to enhance the performance of the base YOLO model, as illustrated in Figure 3c. In the task of classifying rat behavior images, the integration of attention mechanisms is particularly crucial. Discriminating rat behavior often relies on subtle movements or morphological changes, which can be obscured by background noise or interference from irrelevant regions. These two modules aim to optimize the feature extraction process from different dimensions to improve classification performance.

The channel attention mechanism focuses on the channel dimension of the feature maps. It utilizes global pooling operations (such as average or max pooling) to extract global context information for each channel. Then, a lightweight perception module assigns weights to each channel, highlighting the contribution of the key channels. On the other hand, the spatial attention mechanism concentrates on the spatial dimension of the feature maps. By merging information across the channel dimension (such as taking the average or maximum value), it generates a 2D feature map and calculates a spatial attention map through small convolutional layers, emphasizing the significant areas in the image. Channel attention helps the model focus on high-level semantic features related to behavioral patterns, while spatial attention can automatically locate the key areas in the image, such as the rat’s paws, head, or prominent parts of the body. The combination of CBAM and CSPM modules, which employ different designs of channel and spatial attention, allows the network to enhance feature representation at both the channel and spatial levels. These attention mechanisms enable the YOLO model to capture global context while precisely locating the local regions where the behavior occurs, significantly improving classification accuracy and robustness. Thus, this design is better suited to extract discriminative features from rat behavior images, effectively enhancing the classification capability for complex behavioral patterns. Finally, the classification results of each video frame are statistically analyzed (Figure 3d), and the most frequently occurring category is chosen as the classification result for the entire video clip. This approach enables more robust video classification, reducing the impact of single-frame classification errors on the overall classification of the video clip.

### 2.7. Audio Recognition of Teeth Chattering Based on Mel Spectrogram and ResNet-SE

**Figure 4:**
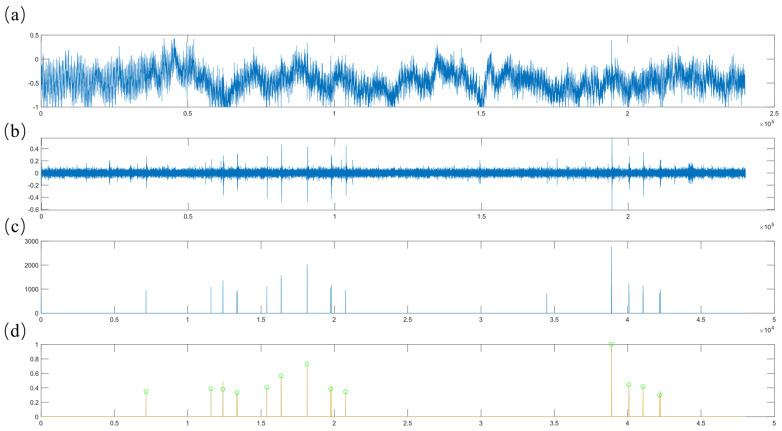
(**a**) The original audio signal. (**b**) The filtered signal. (**c**) The signal after extracting distinct, intermittent sound segments. (**d**) The signal after identifying continuous values. Cyan-colored circles on the Figure 4d indicate initial screening markers for teeth chatter signals.

Teeth chattering exhibits acoustic characteristics that are closely aligned with environmental sounds, such as background noise, and typically has a brief duration. In this study, inspired by the environmental audio dataset ESC-50 [61], we segment continuous recordings into 5 s clips for processing (Figure 4a). The original audio signal clips contain substantial background noise, necessitating the application of median filtering and bandpass filtering to suppress unwanted noise components (Figure 4b). Subsequently, we compute the upper envelope of the filtered signal and set all data points below the mean of this envelope to zero. This step effectively filters out the majority of signal segments with minimal acoustic activity. Given that teeth chattering consists of short, intermittent sounds, densely packed segments of the signal can interfere with the identification of continuous vocalization points. To mitigate this, we downsample the signal and compute the absolute value of the signal gradient to eliminate excessively dense segments. Following these steps, the algorithm normalizes the signal and utilizes a threshold to remove inconspicuous sounds, resulting in a preprocessed audio signal. Experimental results indicate that teeth chattering produces distinct, crisp sounds that appear intermittently over short periods as illustrated in Figure 4c. In the time-domain signal, this corresponds to a characteristic short-term multi-peak waveform. Therefore, if the preprocessed signal contains three or more closely spaced peaks, the system records the corresponding time intervals to facilitate subsequent feature extraction and audio classification, as illustrated in Figure 4d.

**Figure 5:**
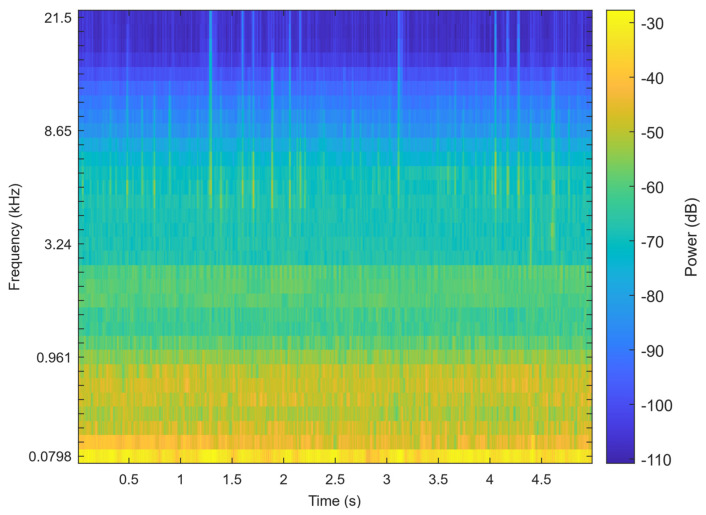
The Mel spectrogram of the signal.

**Figure 6:**
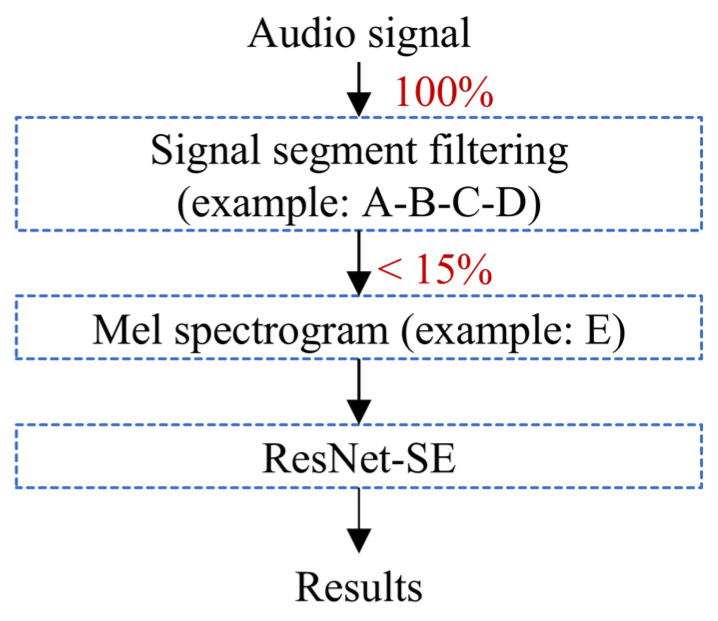
The workflow to identify teeth chattering.

ResNet-SE is effective in learning hierarchical representations of audio from features like Mel spectrograms (Figure 5), which can be further utilized for the classification of teeth chattering audio. The residual structure (ResNet) allows the network to learn the residual mappings between the input and output, thereby simplifying the training of deeper networks. The Squeeze-and-Excitation (SE) module explicitly models the inter-channel relationships, enhancing the network’s selective attention to audio features. This design maintains high performance while also ensuring computational efficiency. The complete processing workflow for chatter audio is illustrated in the Figure 6.

## 3. Results

We summarize the main outcomes of applying MWB_Analyzer, an automated system for detecting morphine withdrawal behaviors in rats. The system combines multi-angle video and audio analysis to provide objective, real-time assessments without manual scoring. Our results show that MWB_Analyzer can accurately identify key withdrawal behaviors, such as wet-dog shakes and scratching (both with over 95% accuracy), and effectively classify more complex behavioral patterns using a deep learning model with over 94% accuracy. Importantly, the system operates smoothly on edge devices, enabling practical use in routine preclinical studies. By applying clear time thresholds and a new scoring scheme, the system helps quantify dose-dependent and time-limited withdrawal symptoms, supporting more reliable and standardized behavioral evaluation.

### 3.1. Analysis of Withdrawal Behavior Recognition Metrics

The behavior recognition algorithm based on one-dimensional difference frame signals converts the inter-frame differences in the original video into a simplified one-dimensional signal, significantly reducing data volume while retaining key behavioral information. This method combines basic signal processing with manual feature extraction to efficiently and rapidly identify wet-dog shakes and scratching behaviors. Experiments show that the recognition accuracy for wet-dog shakes is 95.5% while scratching achieves an accuracy of 95.8%.

**Figure 7:**
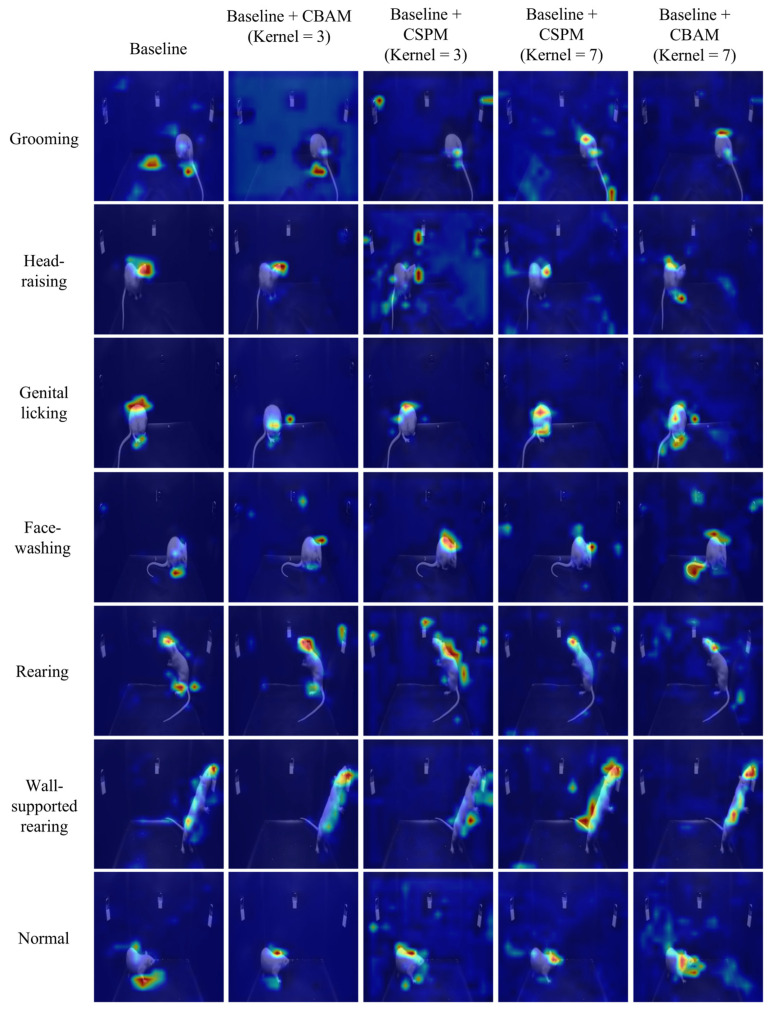
Visualization analysis based on Grad-CAM for the last convolutional layer is used to interpret the model’s prediction decisions. The overlaid pseudo-color heatmap (Grad-CAM) highlights the model’s key regions of interest, with red indicating high activation values (major contribution to prediction) and blue denoting low activation values (minor contribution).

In deep learning models, Grad-CAM (Gradient-weighted Class Activation Mapping) is commonly used to generate class-specific heatmaps to explain the model’s prediction decisions [53]. When selecting the application point for Grad-CAM, we apply it to the last convolutional layer near the classification layer. This choice is made because the feature maps of convolutional layers combine spatial and semantic information, while fully connected or flattened layers in the classification head lose spatial information, rendering Grad-CAM visualizations meaningless. Specifically, the last convolutional layer can be considered a “bottleneck” layer whose output feature maps possess not only high-level semantic abstractions but also provide class-relevant importance information through backpropagated gradients. This enables Grad-CAM to generate class-specific heatmaps with spatial resolution, highlighting the model’s attention regions in the input data, as shown in Figure 7. Through the analysis of Grad-CAM heatmaps, we observed that the “Baseline + CSPM (Kernel = 3)” model demonstrated significant advantages. Its heatmaps accurately focused on the critical regions of the target behavior, such as the head or other key parts of the experimental rats, while maintaining high temporal consistency. This ensures the stable detection of target behaviors across the time dimension. Moreover, the model excelled in handling small-scale movements, further showcasing its robustness and precision in behavioral analysis. A more detailed analysis can be found in Supplementary Materials S3.2.

We constructed a dataset of 10,588 video key frames for YOLO image classification, with the training, validation, and test sets split in a 6:2:2 ratio. We selected the basic YOLOv5 classification model as the baseline model. We trained and tested the baseline model along with five other modified models. The training details can be found in Supplementary Materials S2. The detailed test results are provided in Supplementary Materials S3. In practice, behavioral classification is equivalent to classifying video clips. We built a dataset of 2647 behavioral video clips, with the training, validation, and test sets split in a 6:2:2 ratio. Again, we chose the basic YOLOv5 classification model as the baseline model. We trained and tested the baseline model and five modified models for video classification. The specific test results are shown in Table 1. In conjunction with the heatmap in Figure 7, we observed that the “Baseline + CSPM (Kernel = 3)” model performed best on both key frame and video clip tasks, achieving a 94.2% top-1 classification accuracy. Therefore, we selected this model as the application model and deployed it on the edge computing platform (Orange Pi devices) for testing. Despite the deployment model being reduced to FP16 (half-precision floating point), the classification accuracy on the test set only decreased from 94.2% to 93.6%, still successfully completing the behavior classification task. The model deployed on the RK3588 platform (Orange Pi device) achieved over 40 FPS in video classification tasks, demonstrating sufficient performance for real-time processing.

In this system, we collected 87 segments of teeth chattering audio and 173 segments of other audio sounds, converting them into Mel spectrograms for classification training with the ResNet-SE model. Audio data collected using a dual-microphone setup were processed using logical concatenation methods to ensure the completeness of the recognition results. On a test set of 10 groups, the algorithm achieved an accuracy rate of 92.7%, meeting the experimental requirements. We tested the filtering performance of this method using 20 audio segments, each approximately 30 min long. On average, it successfully filtered out 86.4% redundant data, leaving audio segments that all contained short intermittent sounds. The complete recognition process for a 5 s audio segment took approximately 0.5 s, while the average processing time for 30 min of audio was about 49 s, balancing recognition accuracy and efficiency.

### 3.2. Exploring Behavioral Assessment Schemes for Morphine Addiction

Due to drug stimulation, four rats in the treatment group died, resulting in a final dataset of 11 treated rats and 3 control rats. The hardware system operated stably throughout the data collection process, requiring only one operator to efficiently gather audio and video data from the 15 rats.

Stereotypic behaviors are defined as repetitive, monotonous, and abnormal actions performed by an animal over a certain period. In rodents, these behaviors include repetitive grooming, sniffing, head bobbing, and circling movements [54]. We selected more objective and visually quantifiable metrics from previous studies, which are easier to analyze using visual methods. Therefore, we selected eight behaviors as the evaluation criteria, including wet-dog shakes, scratching, rearing, face washing, grooming, head-raising, genital licking, and teeth chattering. The term “repetition” in the context of stereotypic behavior lacks a quantifiable definition. The question of how long a behavior must persist to be included in statistical counts is worth considering. As noted in the scoring criteria outlined in the introduction, manual counts often overlook this detail. This system incorporates a time threshold to filter automatic recognition results. By experimenting with different thresholds, a more distinct and objective definition of behavior can be established.

A significant advantage of MWB_Analyzer compared to human statistics is its ability to quantify behaviors that exceed a specific time threshold. We have statistically analyzed the results of various behaviors under different time thresholds. The experimental results in Supplementary Materials S4.1 seem to indicate that rat withdrawal behavior appears to last longer compared to normal behavior.

Wet-dog shakes, rearing, and stereotypic behaviors serve as the counting metrics. In contrast, teeth chattering is treated as a non-counting metric, requiring assessment every 5 min to determine its occurrence and score accordingly. When calculating scores for a specific behavior, the occurrences for each animal are first tallied, and the average for each experimental group is computed as the final score. To provide a clearer distinction for teeth chattering, the occurrences of this behavior are also recorded. At this point, a new comprehensive scoring method has been established, as shown in Table 2 below.

### 3.3. Comprehensive Scoring Results Based on Our Proposed Evaluation Scheme

**Figure 8:**
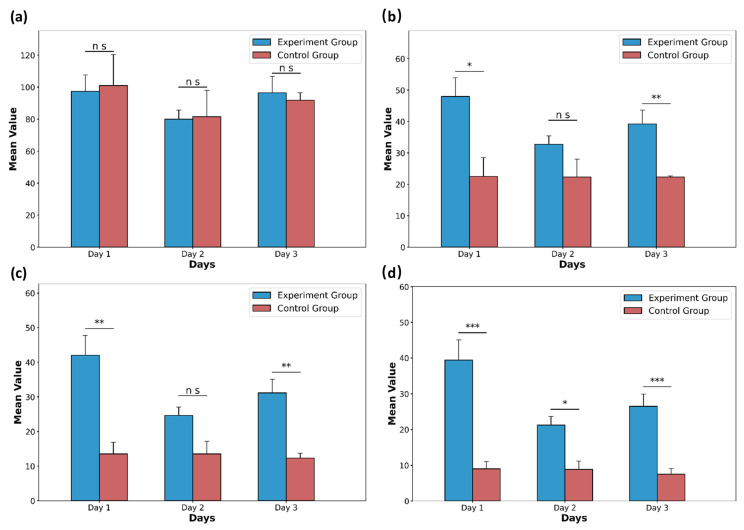
The comprehensive mean scores of the experimental group (*n* = 11) and the control group (*n* = 3) for the first three days of rat withdrawal under different time thresholds. (**a**) Comprehensive scores of withdrawal behavior when the time threshold is 1 s. (**b**) Comprehensive scores of withdrawal behavior when the time threshold is 4 s. (**c**) Comprehensive scores of withdrawal behavior when the time threshold is 7 s. (**d**) Comprehensive scores of withdrawal behavior when the time threshold is 10 s. Data are presented as the mean ± SD. *p* values were calculated by two-tailed, unpaired *t* test in each case, and ns indicates values that are not significant. * *p* < 0.05, ** *p* < 0.01, and *** *p* < 0.001.

Following the criteria outlined in Table 2, comprehensive scores were assigned to several distinguishing behaviors. Figure 8 illustrates the comprehensive scores of the experimental group and the control group for the first three days of rat withdrawal under different time thresholds. Figure 8d demonstrates differences between the experimental group and the control group on day 1 to day 3. This seems to indicate that the rat withdrawal behavior appears to last longer compared to normal behavior. These results indicate that the chosen metrics can effectively reflect the distinctions between the treatment and control groups in the morphine spontaneous withdrawal model in rats, further validating the efficacy of the proposed system.

### 3.4. Experimental Study on Withdrawal Behaviors at Different Morphine Doses

We further conducted experiments to evaluate the sensitivity of our newly developed experimental apparatus (MWB_Analyzer) and the proposed assessment protocol. Rats were divided into three groups: a high-dose group (*n* = 7), a low-dose group (*n* = 10), and a control group (*n* = 4). Behavioral withdrawal scores were recorded daily and analyzed using one-way ANOVA followed by Tukey’s multiple comparisons test.

**Figure 9:**
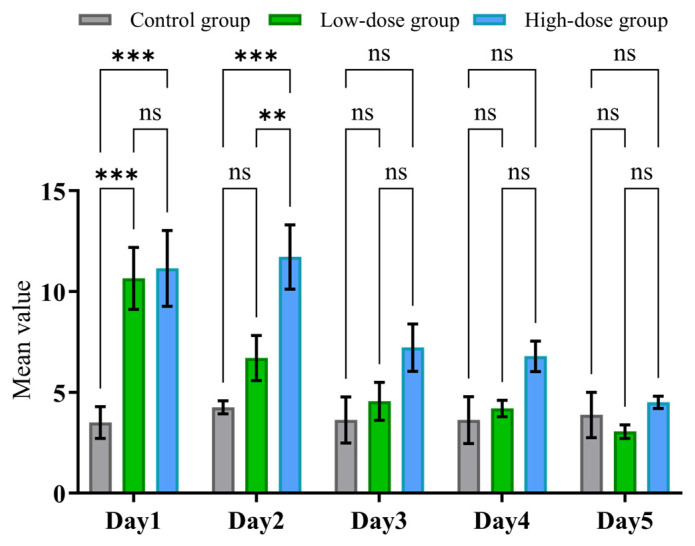
Behavioral withdrawal symptoms were evaluated daily for 5 consecutive days after discontinuation of drug administration. Rats were assigned to three groups: control (*n* = 4), low-dose (*n* = 10), and high-dose (*n* = 7). Data are presented as the mean ± SD. *p* values were calculated by one-way ANOVA followed by Tukey’s multiple comparisons test, and ns indicates values that are not significant. ** *p* < 0.01, and *** *p* < 0.001.

As illustrated in Figure 9, on day 1, the high-dose group exhibited a significant increase in withdrawal behavior scores compared to both the low-dose and control groups (** *p* < 0.001 vs. both groups). The low-dose group also showed significantly higher scores than the control group (* *p* < 0.01), indicating a dose-dependent withdrawal effect immediately following cessation. By day 2, this trend persisted: the high-dose group continued to show significantly elevated scores compared to both other groups (** *p* < 0.001), while the low-dose group again differed significantly from the controls (* *p* < 0.01). These findings suggest sustained withdrawal symptoms at higher dosages. However, from day 3 onward, no statistically significant differences were observed among the three groups (all comparisons marked as “ns”), suggesting a resolution or normalization of behavioral withdrawal symptoms within 72 h post-discontinuation.

These results collectively demonstrate that the severity of withdrawal behaviors is dose-dependent and time-limited, with prominent symptoms occurring during the first two days of withdrawal, particularly in the high-dose group. The resolution of symptoms by day 3 may reflect a rapid adaptation or recovery in the rodent model.

### 3.5. Summary and Implications

Overall, MWB_Analyzer successfully delivers accurate, objective, and efficient assessments of morphine withdrawal behaviors. The system demonstrates clear advantages over manual observation by automating data collection and analysis, reducing subjective bias, and supporting dose–response evaluation. These results highlight its potential as a valuable tool for standardizing preclinical studies of drug dependence and improving the reliability of behavioral pharmacology research.

## 4. Discussion

This study presents a significant advancement in the objective, automated, and user-friendly assessment of morphine withdrawal behaviors in rodent models. The MWB_Analyzer system addresses the key limitations of traditional manual observation—such as subjectivity, labor intensity, and variability between scorers—and provides a practical solution tailored for experimental pharmacologists and toxicologists.

From the user’s perspective, the system offers several clear advantages:

(1) Easy Integration into Standard Workflows: MWB_Analyzer combines multi-angle video and full-scene audio capture in a fully automated manner. Its decentralized design (based on three compact Orange Pi units) requires no technical expertise for setup or operation. Once installed, the system runs autonomously, providing synchronized behavioral recordings without disrupting normal experimental procedures.
(2) Substantial Reduction in Manual Workload Through Automation: The system leverages automatic data filtering and prioritization of key views (e.g., top view for locomotion, side view for postural signs) to reduce redundant data by over 95%. This optimization significantly lowers the computational load, allowing real-time behavior recording and analysis to be performed efficiently on compact edge devices. By automating both data acquisition and analysis, MWB_Analyzer minimizes the need for manual scoring and video review, enabling researchers to obtain objective results with minimal hands-on effort.
(3) Enhanced Objectivity and Reproducibility: MWB_Analyzer achieves high classification accuracy (over 94% for video-based and 92% for audio-based detection of withdrawal behaviors) using a refined machine learning model. Unlike traditional scales such as Gellert and Holtzman [37], the system eliminates subjective judgment, ensuring consistent and unbiased assessments across experiments and operators. This is especially valuable for detecting subtle dose–response differences that might be missed in manual scoring. The system’s real-time analysis and precise behavior recognition facilitate more reliable dose–response evaluations, improved standardization across studies, and higher throughput for preclinical drug screening. By reducing human variability, MWB_Analyzer strengthens the statistical power of pharmacodynamic assessments and enhances confidence in drug efficacy and safety evaluations.
(4) Broad Applicability and Flexibility: While optimized for morphine withdrawal, the system’s modular design allows easy adaptation to other models of dependence (e.g., fentanyl, alcohol). Its architecture also supports studies on drug delivery methods, pharmacokinetics, and systemic effects, making it a versatile tool for a wide range of preclinical research applications.

In summary, MWB_Analyzer not only represents a technical innovation but also provides practical, tangible benefits for researchers, saving time, improving data quality, and facilitating more robust conclusions in addiction pharmacology and beyond.

## 5. Conclusions

In this study, we developed MWB_Analyzer, an innovative and practical system for the automated detection of morphine withdrawal behaviors in rats. The system overcomes the key limitations of traditional manual observation by providing objective, consistent, and high-throughput behavioral assessments. By integrating real-time multi-angle video and audio analysis on compact edge devices, MWB_Analyzer enables efficient data capture and analysis without the need for specialized technical expertise or high-end hardware. Through intelligent data reduction—processing only the most behaviorally relevant 5% of frames—the system ensures smooth real-time operation while maintaining high accuracy (over 94% for video-based behaviors, and over 92% for audio-based signals). This automation minimizes manual workload, eliminates subjective bias, and allows experimental pharmacologists and toxicologists to obtain reproducible dose–response data with greater confidence. Importantly, MWB_Analyzer’s modular and flexible design makes it easy to adapt to other models of dependence (e.g., fentanyl, alcohol) and broader preclinical applications, supporting more reliable drug screening, neuropharmacological research, and evidence-based regulatory evaluations. By replacing manual scoring with a user-friendly, high-fidelity platform, this work provides a valuable tool that enhances both scientific rigor and operational efficiency in addiction research and related fields.

## Figures and Tables

**Table 1:** Testing results of different improved models and the optimal model’s performance on RK3588.

	Criterion	Baseline	Baseline + CBAM (Kernel = 3)	Baseline + CSPM (Kernel = 3)	Baseline + CSPM (Kernel = 7)	Baseline + CBAM (Kernel = 7)	RK3588 (Baseline + CSPM (Kernel = 3))
Grooming	Precision	**0.936**	0.912	0.912	0.928	0.928	0.904
	Recall	0.860	0.832	0.950	0.885	**0.959**	0.950
	F1	0.897	0.870	0.931	0.906	**0.943**	0.926
Head-raising	Precision	0.977	**0.989**	**0.989**	0.977	0.977	**0.989**
	Recall	0.966	0.966	0.966	**0.977**	**0.977**	0.966
	F1	0.971	**0.977**	**0.977**	**0.977**	**0.977**	**0.977**
Normal	Precision	0.879	0.894	**0.924**	0.909	**0.924**	**0.924**
	Recall	0.841	0.881	**0.924**	0.870	0.871	**0.924**
	F1	0.859	0.887	**0.924**	0.889	0.897	**0.924**
Rearing	Precision	**0.915**	0.872	0.894	0.809	0.830	0.851
	Recall	0.977	0.854	**1.000**	**1.000**	**1.000**	**1.000**
	F1	0.945	0.863	**0.994**	0.894	0.907	0.920
Genital licking	Precision	0.831	0.803	**0.958**	0.887	0.972	**0.958**
	Recall	0.952	0.934	0.919	**0.984**	0.896	0.932
	F1	0.887	0.864	0.938	0.933	0.932	**0.944**
Wall-supported rearing	Precision	0.990	0.930	0.990	**1.000**	**1.000**	0.990
	Recall	**0.961**	0.949	**0.961**	0.926	0.943	0.943
	F1	**0.975**	0.939	**0.975**	0.962	0.971	0.966
Face-washing	Precision	0.769	0.821	**0.872**	0.821	0.821	**0.872**
	Recall	0.909	**0.914**	0.829	0.842	**0.914**	0.791
	F1	0.833	**0.865**	0.850	0.831	**0.865**	0.829
All	Top-1	0.918	0.901	**0.942**	0.923	0.938	0.936

Note: Bold values indicate the maximum within each respective metric category.

**Table 2:** A novel comprehensive scoring method.

Types of Behavior	Scoring Method
Wet-dog shakes	1 point each time recorded
Head-raising	1 point each time recorded
Stereotypic behaviors	0.5 point each time recorded
Teeth chattering	2 points if it occurs every 5 min

## Data Availability

The code and datasets used and analyzed during the current study are available in the GitHub repository at https://github.com/Zhang-Moran/MWB_Analyzer (accessed on 2 July 2025).

## Supplementary Materials

The following supporting information can be downloaded at https://www.mdpi.com/article/10.3390/toxics13070586/s1. Text S1: Details on Synchronous Video Stream Acquisition and Real-time Video Segment Filtering; Text S2: Details in Model Training; Text S3: Details in Results of Image Classification; Text S4: Details in behavior statistics; Table S1: Test results of different improved YOLO models on the image test set. Figure S1: Visualization analysis based on Grad-CAM for the last convolutional layer is used to interpret the model’s prediction decisions. Figure S2: Results of grooming behavior under different time durations. A. Results of behavior lasting > 1 s. B. Results of behavior lasting > 4 s. C. Results of behavior lasting > 7 s. D. Results of behavior lasting > 10 s. E. Results of behavior lasting > 13 s. Figure S3: Results of head-raising behavior under different time durations. A. Results of behavior lasting >1 s. B. Results of behavior lasting > 4 s. C. Results of behavior lasting > 7 s. D. Results of behavior lasting > 10 s. E. Results of behavior lasting > 13 s. Figure S4: Results of rearing behavior under different time durations. A. Results of behavior lasting > 1 s. B. Results of behavior lasting > 4 s. C. Results of behavior lasting > 7 s. D. Results of behavior lasting >10 s. Figure S5: Results of genital licking behavior under different time durations. A. Results of behavior lasting > 1 s. B. Results of behavior lasting > 4 s. C. Results of behavior lasting > 7 s. D. Results of behavior lasting > 10 s. E. Results of behavior lasting >13 s. Figure S6: Results of wall-supported rearing behavior under different time durations. A. Results of behavior lasting > 1 s. B. Results of behavior lasting > 4 s. C. Results of behavior lasting > 7 s. D. Results of behavior lasting > 10 s. Figure S7: Results of face-washing behavior under different time durations. A. Results of behavior lasting > 1 s. B. Results of behavior lasting > 4 s. C. Results of behavior lasting > 7 s. D. Results of behavior lasting > 10 s. E. Results of behavior lasting > 13 s. Figure S8: A. results of scratching behavior. B. Results of wet-dog shaking behavior. Figure S9: Results of teeth chattering behavior.
