# Supplementary material for: MWB_Analyzer: An Automated Embedded System for Real-Time Quantitative Analysis of Morphine Withdrawal Behaviors in Rodents
Source: Toxics. 2025 Jul 14;13(7):586. doi: 10.3390/toxics13070586 (PMC12298654; doi:10.3390/toxics13070586)
Supplement: Supplementary file 1 [file toxics-13-00586-s001.zip › toxics-3673823-supplementary.pdf]

Supplementary Information for

Article

MWB_Analyzer: An Automated Embedded System for Real-Time Quantitative Analysis of Morphine Withdrawal Behaviors in Rodents

Moran Zhang [1,2,†], Qianqian Li [3,†], Shunhang Li [1,†], Binxian Sun [1], Zhuli Wu [1], Jinxuan Liu [4], Xingchao Geng [3,*] and Fangyi Chen [1,2,*]

[1] Department of Biomedical Engineering, Southern University of Science and Technology, Shenzhen 518055, China;
[2] Guangdong Provincial Key Laboratory of Advanced Biomaterials, Southern University of Science and Technology, Shenzhen 518055, China
[3] National Center for Safety Evaluation of Drugs (NCSED), National Institutes for Food and Drug Control, Beijing 102629, China;
[4] Shenzhen Giant (Ju'An) Technologies Co., Ltd., Shenzhen 518000, China;

* Correspondence: gengxch@nifdc.org.cn (X.G.); chenfy@sustech.edu.cn (F.C.)
† The authors contributed equally to this work.

This PDF file includes the following:

Figures S1–S9
Tables S1

S1 Details on Synchronous Video Stream Acquisition and Real-time Video Segment Filtering

The design of the video stream acquisition and dimensionality reduction module represents one of the core components of the behavioral video analysis system. Its objective is to ensure the synchronous acquisition of high-quality video data from multiple viewpoints while also enabling efficient data processing and dimensionality reduction. In studies of rodent behavior, researchers are often confronted with massive volumes of video data. The selection of video segments directly affects the efficiency and accuracy of subsequent behavioral recognition and data analysis. In this research, we propose an efficient video segment selection method based on signal processing and viewpoint selection. This method is capable of reducing the video data volume by approximately 90%, thereby laying the foundation for real-time processing.

S1.1 Synchronous Video Stream Acquisition and Video Data Dimensionality Reduction

S1.1.1 Video Stream Acquisition Based on RK3588

To capture behavioral features comprehensively, we designed and implemented an efficient multi-threaded video capture and processing system using five USB cameras to acquire multi-angle video data. The system adopts FFmpeg as the core framework for video capture and decoding to support the MJPEG stream format from USB cameras. Initially, camera devices are registered to ensure that FFmpeg can correctly identify the input sources. Subsequently, the video streams are opened and stream information is extracted to confirm compatibility between the input device parameters and the stream format. Once the video streams have been captured, the system selects the target stream for decoding. To achieve this, the code iterates over all input streams to identify the video stream index and then initializes the corresponding decoder context. The system dynamically recognizes the specific encoding format of the camera stream and allocates the required memory resources for the decoder. During the video frame acquisition process, a timestamp-based frame synchronization mechanism is also implemented. By using both presentation timestamps and decoding timestamps, each frame is annotated with a time offset relative to the first frame, thereby ensuring consistency in the temporal order. This synchronization mechanism guarantees that subsequent processing and streaming adhere strictly to the frame's timeline. The introduction of multi-threading is key to the system's efficient operation. To ensure orderly concurrency and consistent data integrity, the system employs a producer–consumer model, whereby one thread is responsible for frame capture and production, and multiple consumer threads are tasked with frame analysis, encoding, and streaming. The threads are decoupled through a buffering queue, allowing for flexible task distribution and parallel processing.

S1.1.2 Multi-camera Video Stream Synchronization Based on External Triggering

High-precision synchronization of video streams is a critical factor in multi-view behavioral analysis, particularly for accurately mapping the temporal and spatial relationships of complex behavioral events. To address the issues of temporal offset and data drift between multiple cameras, we propose a synchronization scheme that integrates hardware trigger signals with software control. This scheme is designed to ensure that all cameras initiate acquisition based on a unified time reference and continuously record multi-view behavioral video data with spatiotemporal consistency.

The external-trigger-based video stream synchronization relies on hardware signals (e.g., pulse signals, optical triggers, or voltage level changes) as the trigger source, in combination with high-precision timestamping and software-level data calibration, to achieve synchronized control across multiple cameras. The core steps are as follows:

1. Trigger Signal Description. A dedicated signal generator is employed to produce a synchronous trigger signal, which may take the form of a square wave, voltage level change, or optical signal. In this study, an optical trigger (scene brightness change) is adopted. The trigger signal is distributed to all cameras via a signal distributor to ensure that every device receives the trigger pulse simultaneously. When the infrared lamp is off, nearly all pixel values are close to zero, resulting in a near-black image; when the infrared lamp is activated, the pixel values in certain regions (e.g., reflective areas or target objects) rapidly increase. Hence, the trigger moment is defined as follows:

$$P(t) = \sum_{i=1}^{M} \sum_{j=1}^{N} I_{i,j}(t)$$

$$t_{trigger} = \min\{t \mid P(t) > P_{threshold}\},$$

where $P(t)$ denotes the sum of all pixel values (or the average pixel value over a specific ROI) in the image;

$I_{i,j}(t)$ represents the value of the pixel located at (i, j);

$P_{threshold}$ is a preset threshold for pixel change, used to detect the activation of the infrared lamp.

To avoid false triggering due to environmental noise, threshold criteria or a temporal window filter can be applied to the pixel variation. For instance, one may require that for a continuous interval Δt, the pixel values continuously exceed the threshold $P_{threshold}$:

$$t_{trigger} = \min\{t \mid \forall t' \in [t, t + \Delta t],\ P(t') > P_{threshold}\}.$$

2. Modeling of the Acquisition Start Time. After receiving the trigger signal, each camera initiates its acquisition process via its hardware control interface (e.g., GPIO, trigger interface, or external clock input). This hardware-triggered approach circumvents delays and control logic deviations inherent in software control. Let the camera's frame sampling rate be f_{camera} and the inter-frame interval be $T_{camera} = \frac{1}{f_{camera}}$. The acquisition time t_n for the nth frame is aligned with the trigger signal $t_{trigger}$ and is defined as

$$t_n = t_{trigger} + n \cdot T_{camera}, \quad n = 0, 1, 2, \ldots$$

where $t_{trigger}$ represents the trigger moment at the transition from lamp-off to lamp-on;

n denotes the nth frame acquired by the camera;

T_{camera} is the frame time interval.

3. Alignment Conditions for Multi-camera Synchronization. For multi-camera synchronization, the trigger signal $t_{trigger}$ must be sent uniformly to all cameras. Assuming that all cameras share the same sampling frequency, let the acquisition time for camera i be $t_{n,i}$; the synchronization condition then is

$$t_{n,1} = t_{n,2} = \cdots = t_{n,k} = t_n,$$

where k is the number of cameras.

If certain cameras exhibit startup delays Δt_i, it is necessary to calibrate these delays such that

$$\Delta t_i = 0 \quad (\forall i).$$

This calibration is typically completed through hardware synchronization circuits or post-processing software alignment.

4. Timestamp Alignment and Post-correction. Building upon hardware triggering, high-precision timestamps are assigned to each video frame to ensure the precise matching of acquisition times. Additionally, post hoc alignment of frame sequences further eliminates minor deviations caused by sampling frequency drift. Although the use of optical triggers synchronizes acquisition, cameras may still record each frame with a slight error ϵ_i. These errors can be corrected by adjusting the frame timestamps:

$$t_{n,i}^{aligned} = t_{n,i} - \epsilon_i,$$

where the error ϵ_i can be estimated by cross-comparing the intensity variations recorded by all cameras, such as by comparing the frame indices or timestamps at lamp activation.

In summary, the brightness-change trigger-based synchronization method is highly suitable for experiments that require high-precision multi-view recording. By marking the behavioral timeline at the moment of lamp activation, a unified starting point for the video streams is established. Its advantages lie in the fact that hardware triggering eliminates delays and jitters associated with traditional software control—often arising from operating system scheduling—in favor of microsecond-level time synchronization. The simple, reliable trigger signal, coupled with natural integration via brightness changes, obviates the need for complex cabling arrangements and minimizes hardware interference. Ultimately, the combination of hardware triggering and software timestamping provides a robust basis for subsequent multi-view data alignment and spatiotemporal analysis, significantly enhancing the precision and stability of multi-camera video stream synchronization and offering a solid data foundation for complex behavior analysis.

S1.1.3 Video Data Dimensionality Reduction

In the data dimensionality reduction phase, we first employ an inter-frame differencing technique to compute the pixel intensity differences between successive frames, thereby extracting the change information from each frame of the video sequence. Mathematically, the inter-frame difference is expressed as

$$D_t(i, j) = |I_t(i, j) - I_{t-1}(i, j)|\,,$$

where $D_t(i, j)$ is the difference value of the pixel at (i, j) in the tth frame;

$I_t(i, j)$ is the pixel intensity at position (i, j) in the tth frame;

$I_{t-1}(i, j)$ is the pixel intensity at the same position in the $(t-1)$th frame.

By statistically processing the difference values of the entire frame, we can generate an overall dynamic signal representation for each frame:

$$S_t = \sum_{i=1}^{M} \sum_{j=1}^{N} D_t(i, j),$$

where S_t denotes the overall change intensity of the tth frame;

M is the width of the frame;

N is the height of the frame.

Inter-frame differencing effectively removes static background information, retaining only the dynamic characteristics of the regions of interest. This highlights the key behavioral regions, significantly enhancing the system's ability to detect the target behavior. Subsequently, based on the time-series data $\{S_1, S_2, \ldots, S_T\}$ generated by the frame differencing, we construct a complete dynamic signal sequence, thereby laying the groundwork for further behavioral analysis. This dimensionality reduction process not only markedly reduces the redundant information in the original video data but also ensures that the key dynamic features required for scientific and reliable behavioral analysis are preserved.

To further enhance the quality and robustness of the reduced data, we introduce median filtering as a noise suppression technique, targeting the removal of sharp peaks caused by outliers. The median filtering is mathematically expressed as

$$\hat{S}_t = \text{median}(S_{t-k}, S_{t-k+1}, \ldots, S_{t+k}),$$

where $\hat{S}_t$ is the filtered signal of the tth frame;

$2k + 1$ denotes the size of the filter window.

The benefit of median filtering is that it effectively eliminates the interference of spurious sharp peaks (i.e., high, narrow peaks) due to noise while preserving the overall trend of the changes. The reduced data is subsequently stored as a compact time-series signal, which not only optimizes the use of storage and computational resources but also provides a high-quality input for the subsequent extraction and analysis of behavioral features. By effectively retaining key dynamic features and eliminating noise interference, the reduced data plays a crucial role in dynamic behavior modeling and recognition. In particular, for tasks involving time-series analysis and the classification of dynamic behaviors, this dimensionality reduction process provides a robust foundation for capturing the temporal dependencies and spatial variations of the behavior, thereby significantly improving the overall performance of the algorithms.

S1.2 Video Segment Extraction Incorporating Motion Information Based on Signal Processing

S1.2.1 Motion Information Enhancement Based on Multi-frame Data Fusion and Exponentially Weighted Moving Average

Multi-frame data fusion is a signal processing technique that integrates information from multiple frames to enhance signal quality, accuracy, or reliability. Its fundamental idea is to leverage redundant and complementary information across different temporal frames to reduce noise interference and strengthen the representation of useful signal features. In the signal processing process, multi-frame fusion not only allows for a comprehensive analysis of the target signal from multiple temporal perspectives but also effectively smooths out random noise, thereby improving the signal-to-noise ratio. Particularly in dynamic scenes, the fusion of multi-frame data aids in capturing temporal variations of the target signal, subsequently enhancing its feature contrast or energy distribution. Specifically, for a difference signal, a squared-sum fusion method can be employed. This method aggregates the squares of the signals from the previous n frames via a sliding window, optionally taking the square root to maintain consistent dimensions. This is mathematically expressed as

$$S_{\text{fused\_signal}}(i) = \sqrt{\sum_{j=i-n+1}^{i} S(j)^2},$$

where $S(j)$ represents the amplitude of the input signal at the jth frame;

$S_{\text{fused\_signal}}(i)$ denotes the fused signal value at the ith frame.

The squaring operation nonlinearly amplifies strong signal regions, thereby significantly emphasizing the energy distribution of these regions while suppressing noise in weaker regions. This approach emphasizes salient target features without appreciably losing the original structural information of the signal. Multi-frame fusion techniques are widely used in image processing, video processing, and various sensor data analyses (including applications such as target detection, trajectory analysis, and signal recovery), where processing consecutive frames jointly considerably enhances signal reliability and usability.

The Exponentially Weighted Moving Average (EWMA) is a widely applied smoothing technique in time-series analysis, known for assigning greater weight to recent observations. Its theoretical basis is the recursive, weighted average that simultaneously captures short-term fluctuations and smooths long-term trends. The EWMA is given by

$$S_t = \alpha \cdot x_t + (1 - \alpha) \cdot S_{t-1},$$

where S_t is the smoothed value at time t;

x_t is the observation at that time;

α is the smoothing coefficient, with $0 < \alpha \leq 1$.

When α is relatively large, the algorithm becomes more sensitive to recent data, whereas a smaller α produces a smoother response to short-term fluctuations, revealing longer-term trends. In the field of signal processing, this exponentially decaying weighting mechanism makes EWMA particularly suitable for enhancing dynamic features. For example, in the detection of moving targets, applying EWMA to a series of difference frames can effectively suppress the interference of background noise while boosting the signal strength in motion regions, thereby significantly improving the target's visibility. This method seamlessly merges the smoothing properties of time-series analysis with the sensitivity to new information, offering a highly flexible and accurate solution for motion information processing.

S1.2.2 Signal Segment Extraction Based on Signal Envelope and Baseline Correction

Signal envelope extraction based on the Hilbert transform is a common signal processing technique used to analyze the amplitude trends of a signal over time. It is particularly suitable for non-stationary signals such as vibration signals or communication signals. In signal processing, the envelope refers to the smooth curve connecting the local extrema of the signal, thereby reflecting the amplitude variation and overall energy distribution of the signal. The Hilbert transform is the core tool for envelope extraction, representing a signal's amplitude and phase changes by constructing its complex analytic signal. Specifically, the Hilbert transform is defined by the following integral:

$$H[x(t)] = \frac{1}{\pi} \int_{-\infty}^{\infty} \frac{x(\tau)}{t - \tau} \, d\tau,$$

where $x(t)$ is the real-valued signal;

$H[x(t)]$ is the Hilbert transform, which yields the imaginary part that is orthogonal to the original signal.

By combining the original signal $x(t)$ with its Hilbert transform $H[x(t)]$, one can construct the analytic signal

$$z(t) = x(t) + jH[x(t)],$$

which maps the signal onto the complex plane and facilitates subsequent feature extraction.

The signal envelope $A(t)$ is defined as the modulus of the analytic signal. In practice, two methods are typically used for envelope calculation: the "peak" method and the "RMS" method. The "peak" method extracts the envelope as the peak values of the signal; that is, the modulus of the analytic signal yields $A_{\text{peak}}(t)$ and is computed via the Hilbert transform or interpolation of local extrema. In contrast, the "RMS" method computes the root-mean-square envelope, which reflects the local energy trend of the signal rather than merely its amplitude. The RMS envelope is particularly suited for describing the energy distribution of the signal and is expressed as

$$A_{\text{peak}}(t) = \sqrt{x(t)^2 + H[x(t)]^2} = |z(t)| = \sqrt{x(t)^2 + H[x(t)]^2}$$

$$A_{\text{RMS}}(t) = \sqrt{\frac{1}{w} \int_{t-w/2}^{t+w/2} x^2(\tau) \, d\tau}.$$

Here, the RMS envelope not only provides a smooth trend of the local energy changes in the signal but is also robust against noise, making it ideal for analyzing stationary signals or energy variations. Since this study focuses on extracting sustained motion segments, we opt to use the RMS envelope to mitigate the influence of sharp peaks and noise. In physical terms, the analytic signal can be represented in polar form as $z(t) = A(t)e^{j\phi(t)}$, where

$A(t)$ is the amplitude envelope and $\phi(t)$ is the instantaneous phase. This representation decouples the amplitude information from the phase information, thereby facilitating analysis.

Baseline correction is a common signal processing method aimed at eliminating offsets or background noise in order to accurately extract the effective signal. Particularly during envelope analysis, the baseline of a signal may shift due to factors such as device drift or environmental changes. Baseline correction helps recover the true signal characteristics. In this study, we propose a histogram-based baseline correction method, detailed as follows.

First, the core of the baseline correction is to estimate the baseline value by analyzing the distribution characteristics of the signal. The signal $x(t)$ is fed into a histogram calculation function that segments the amplitude range into discrete bins and counts the frequency for each bin. We denote the discrete data points of the signal as $\{x_1, x_2, \ldots, x_n\}$. The histogram yields the frequency counts binCounts$[i]$ for each bin and the corresponding bin edges binEdges$[i]$, which can be computed as

$$\text{binCounts}[i] = \sum_{j=1}^{n} \mathbb{1}\Big(\text{binEdges}[i-1] \leq x_j < \text{binEdges}[i]\Big),$$

where $\mathbb{1}(\cdot)$ is the indicator function that checks whether x_j falls within the interval $[\text{binEdges}[i-1], \text{binEdges}[i])$.

Next, the baseline value is determined by identifying the bin with the maximum frequency; let the index of this bin be maxIdx. The upper edge of this bin is then taken as the signal's baseline:

$$\text{BaselineofEnvelope} = \text{binEdges}[\text{maxIdx}].$$

This value represents the "typical" amplitude level of the signal, which usually corresponds to the stationary part or background noise.

Once the baseline is determined, the signal is corrected relative to this baseline. Specifically, for each sampled point x_i in the signal, if its value is below the baseline plus a predetermined offset (e.g., 1.0), it is regarded as belonging to the background noise or low-amplitude component, and the value is set to zero:

$$\text{if } x_i < \text{BaselineofEnvelope} + \Delta, \text{ then set } x_i = 0,$$

where Δ is a threshold, typically set to 1.0 or adjusted according to practical requirements. This correction effectively eliminates minor fluctuations below the baseline, thereby emphasizing the main features of the signal.

Through this histogram-based baseline correction method, signal offsets can be automatically rectified, which significantly improves the accuracy and reliability of subsequent envelope extraction and feature analysis. The method's advantages lie in its intuitive and flexible approach, making it well suited to handling different types of noise and signal offsets.

S1.3 Video Segment Selection Based on Camera Viewpoint

In this study, we propose an image-processing-based method for camera position recognition. The core of this method is to extract and analyze input images using morphological operations and contour analysis, combined with ellipse fitting, to compute and output the optimal camera position identifier. In practice, the input image is first converted to grayscale and then binarized via thresholding to extract contour information. Subsequently, morphological operations (erosion and dilation) are applied to the binary image to eliminate noise and enhance the contours, thus facilitating more accurate contour extraction. Following this, ellipse fitting is performed based on the extracted contours. By identifying the contour with the largest area, the primary elliptical shape is determined, whose centroid and major axis orientation serve as key parameters for subsequent computations.

Once the ellipse is fitted, the next critical step is to calculate the Euclidean distances from the ellipse's center to the centers of the four image edges (top, bottom, left, and right). By computing these distances, the edge closest to the ellipse center can be identified. To further enhance the accuracy of the positioning, the method also calculates the angle between the ellipse's major or minor axis and the vectors from the ellipse center to the image edges. Specifically, the ellipse's major axis vector is derived from its rotation angle and the length of the major axis. Using a rotation matrix, the angles between the four edge center vectors and the ellipse's minor axis are computed as follows:

$$\theta_{top} = |\text{atan2}(y_{top}, x_{top}) - \text{atan2}(y_{minor}, x_{minor})|$$
$$\theta_{bottom} = |\text{atan2}(y_{bottom}, x_{bottom}) - \text{atan2}(y_{minor}, x_{minor})|$$
$$\theta_{left} = |\text{atan2}(y_{left}, x_{left}) - \text{atan2}(y_{minor}, x_{minor})|$$
$$\theta_{right} = |\text{atan2}(y_{right}, x_{right}) - \text{atan2}(y_{minor}, x_{minor})|\,,$$

where θ_{top}, θ_{bottom}, θ_{left}, and θ_{right} denote the angles between the ellipse's minor axis and the vectors from the ellipse center to the top, bottom, left, and right edge centers, respectively. By comparing these angles, the camera position that is most proximal to the ellipse can be determined.

Finally, by combining the edge distance and angular information, the algorithm outputs a camera position identifier, which is determined based on the edge with the shortest distance and the greatest angular discrepancy. For instance, if the shortest distance corresponds to the top edge, the corresponding angle determines whether the camera position is classified as bottom, left, or right; similar logic applies for the other edges. Throughout the calculation process, floating-point errors are minimized by using a small threshold to avoid misclassification due to precision issues.

This method accurately outputs the camera position identifier by leveraging the geometric relationship between the elliptical shape derived from the image content and the image boundaries.

Algorithm 1: Image Preprocessing and Morphological Operations

Data: Input image $image$
Result: Processed binary image after morphological operations
Step 1: Image Preprocessing;
Convert $image$ to grayscale img_gray;
Apply binary thresholding to img_gray, resulting in binary image $binary\_img$;
Step 2: Morphological Operations (Erosion and Dilation);
Initialize $erosion\_size = 6$;
Initialize $dilation\_size = 5$;
Initialize $num\_iterations = 5$;
for $i = 1$ ***to*** $num\_iterations$ **do**
    Perform erosion on $binary\_img$ with structuring element size $(2 \times erosion\_size + 1)$;
    Perform dilation on the result of erosion with structuring element size $(2 \times dilation\_size + 1)$;
return *Processed binary image after morphological operations*

Algorithm 2: Contour Detection and Ellipse Fitting

Data: Processed binary image after morphological operations
Result: Ellipse parameters of the largest contour
Step 3: Find Contours;
Find contours in the binary image and store them in $contours$ list;
Step 4: Ellipse Fitting and Selection of Largest Contour;
Initialize $max\_area = 0$;
foreach $contour \in contours$ **do**
    if *contour has fewer than 5 points* **then**
        continue;
    Fit an ellipse to $contour$ and get ellipse parameters;
    Calculate $contour\_area$;
    if $area > max\_area$ **then**
        $max\_area \leftarrow area$;
        Set $max\_ellipse$ to the current ellipse;
return $max\_ellipse$

Algorithm 3: Calculate Camera Position

Data: Ellipse parameters $max\_ellipse$, Image size $image.width, image.height$

Result: Camera position identifier $camera\_id$

Step 5: Calculate the Major Axis Vector;
Calculate $center = max\_ellipse.center$;
Calculate $major\_axis\_length = \max(max\_ellipse.size.width, max\_ellipse.size.height)$;
Calculate $angle = max\_ellipse.angle$;
Calculate $major\_axis\_vector = (\cos(angle), \sin(angle)) \times major\_axis\_length/2$;
Step 6: Define Image Edge Centers;
Define $top\_center = (image.width/2, 0)$;
Define $bottom\_center = (image.width/2, image.height)$;
Define $left\_center = (0, image.height/2)$;
Define $right\_center = (image.width, image.height/2)$;
Step 7: Calculate Distance from Ellipse Center to Edge Centers;
Calculate distances to each edge from ellipse center:
top_distance = EuclideanDistance(center, top_center);
bottom_distance = EuclideanDistance(center, bottom_center);
left_distance = EuclideanDistance(center, left_center);
right_distance = EuclideanDistance(center, right_center)
Step 8: Determine the Shortest Distance to an Edge and Select Camera;
Initialize $camera\_id = 0$;
if $top\_distance$ *is the smallest distance* **then**
    Select camera based on angles of other edges;
else if $bottom\_distance$ *is the smallest distance* **then**
    Select camera based on angles of other edges;
else if $left\_distance$ *is the smallest distance* **then**
    Select camera based on angles of other edges;
else if $right\_distance$ *is the smallest distance* **then**
    Select camera based on angles of other edges;
return $camera\_id$

S2 Details on Model Training

The model is trained using a batch size of 16 and a total of 35 rounds of training.

Using the smart optimizer that comes with the YOLOv5 model, with momentum of 0.9 and decay of 5e-5, the learning rate was adjusted using the cosine annealing method with restarting and setting the initial learning rate to 0.001. The iteration epoch before the first restart is five, and each subsequent iteration is twice the number of rounds of the previous iteration.

Using smart cross-entropy loss to measure the difference between training results and the ground truth, we utilized a YOLOv5s pre-trained classification model, which was initially trained on the COCO dataset. The training process was conducted on an NVIDIA M40 GPU with 24GB of VRAM.

S3 Details on Results of Image Classification

S3.1 Test results of different improved YOLO models on the image test set

Tab. S1. Test results of different improved YOLO models on the image test set.

	Criterion	Baseline	Baseline + CBAM (Kernel = 3)	Baseline + CSPM (Kernel = 3)	Baseline + CSPM (Kernel = 7)	Baseline + CBAM (Kernel = 7)
Grooming	Precision	0.85	0.82	0.92	0.88	0.94
	Recall	0.93	0.88	0.92	0.91	0.9
	F1	0.89	0.85	0.92	0.89	0.92
Head-raising	Precision	0.96	0.96	0.95	0.98	0.97
	Recall	0.97	0.97	0.97	0.97	0.97
	F1	0.97	0.97	0.96	0.97	0.97
Normal	Precision	0.85	0.86	0.93	0.85	0.85
	Recall	0.88	0.89	0.92	0.92	0.92
	F1	0.86	0.88	0.92	0.88	0.89
Rearing	Precision	0.97	0.87	0.98	0.98	0.98
	Recall	0.91	0.87	0.84	0.81	0.85
	F1	0.94	0.87	0.91	0.89	0.91
Genital licking	Precision	0.94	0.88	0.9	0.97	0.89
	Recall	0.83	0.79	0.94	0.88	0.95
	F1	0.88	0.83	0.92	0.92	0.92
Wall-supported rearing	Precision	0.96	0.95	0.95	0.93	0.94
	Recall	0.97	0.93	0.98	0.99	0.99
	F1	0.97	0.94	0.96	0.96	0.97
Face-washing	Precision	0.86	0.86	0.82	0.81	0.83
	Recall	0.72	0.78	0.82	0.79	0.77
	F1	0.78	0.82	0.82	0.8	0.8
All	Top-1	0.909	0.886	0.926	0.914	0.923
	Macro Precision	0.911	0.886	0.921	0.913	0.916
	Macro Recall	0.889	0.873	0.911	0.895	0.906
	Macro F1	0.898	0.878	0.915	0.902	0.91
	MCC	0.892	0.863	0.911	0.897	0.908

The head-raising and wall-supported rearing behaviors achieved good results across all models. For grooming and genital licking behaviors, the CSPM (k=3) and CBAM (k=7) models exhibited higher accuracy. Overall, the CSPM (k=3) model achieved the highest top-1 accuracy, while the attention mechanism in the CBAM (k=3) model actually lowered the overall accuracy. Although the baseline model did not incorporate attention enhancement, it still performed well on the similar behaviors of rearing and wall-supported rearing. This suggests that in datasets containing both similar and dissimilar behaviors, the attention mechanism might introduce interference. The accuracy for face-washing was relatively low, likely due to the small number of samples in this category within the dataset. However, the CSPM(k=3) model still achieved a good balance between precision and recall. In terms of macro precision, macro recall, macro F1, and MCC metrics, the CSPM(k=3) model performed the best,

indicating that this model is effective at feature extraction even in imbalanced datasets.

S3.2 Grad-CAM-based Visualization and Model Explanation Details

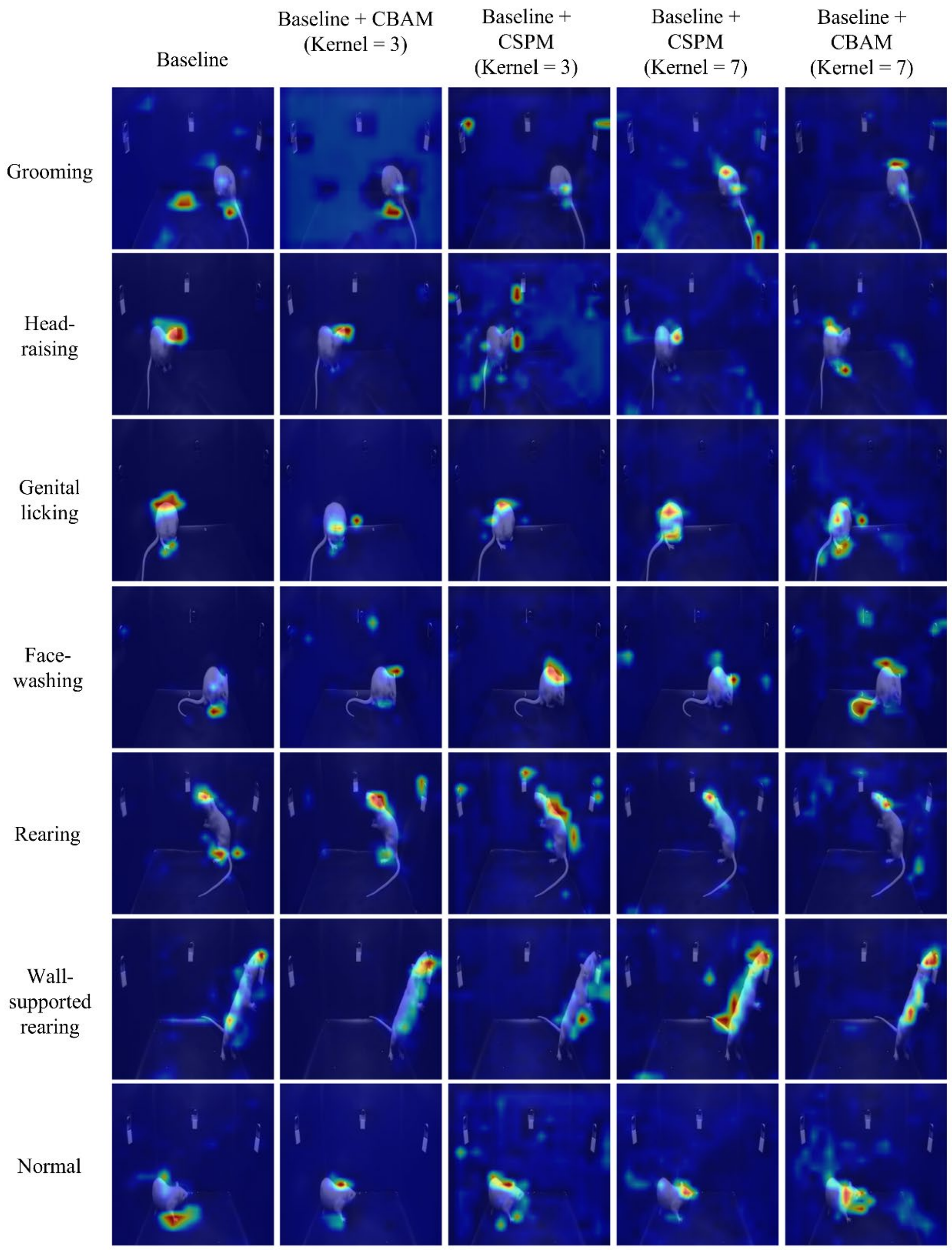

Fig. S1. Visualization analysis based on Grad-CAM for the last convolutional layer is used to interpret the model's prediction decisions.

In head-raising and wall-supported rearing behaviors, all models showed high recognition accuracy. It was observed that in the head-raising behavior, nearly all models focused on the head position. In wall-supported rearing, where the head posture is similar to head-raising, the baseline model, CBAM model, and CSPM (k=7) used both head and body postures to identify the behavior, while CSPM (k=3) focused

more on the interaction between the rat's body and the enclosure.

In grooming and normal behaviors, CSPM (k=3) achieved higher F1 scores, with the precision and recall scores being very close. From the heatmap, it can be seen that in grooming behavior, the model was able to precisely capture the rat's grooming position without being distracted by other body postures (such as the tail or back). In normal behavior, due to the broader range of postures and positions, the model focused more on the rat's body posture and integrated features from the paws to make judgments.

For genital licking and face-washing behaviors, CSPM (k=3) showed higher recognition rates. However, from the heatmap, it was found that the model tended to rely on body posture during the behavior (paying more attention to back curvature) to identify these two behaviors.

Additionally, in the similar behaviors of rearing and wall-supported rearing, the baseline model exhibited higher accuracy. During the recognition process, rearing focused more on the state of the paws and head, while wall-supported rearing focused on the head and body. This might be due to the attention mechanism, which led to a focus on incorrect body postures or interactions with the environment in rearing, causing a decrease in recall for other models.

We found that when using parallel attention modules, the accuracy of the larger kernel was lower than that of the smaller kernel, whereas with serial attention modules, the accuracy of the larger kernel was higher. This may be because, in parallel attention, the smaller kernel can more finely extract spatial information, thereby focusing on more behavior-related information, such as the rat's different postures and environmental states. In serial attention, however, because the activation passes through both channel and spatial attention sequentially, the smaller kernel may mask information from channel attention, leading to attention interference.

S4 Details on behavior statistics

S4.1 Statistics of Various Behaviors in Rats During the First Three Days of Withdrawal at Different Time Thresholds

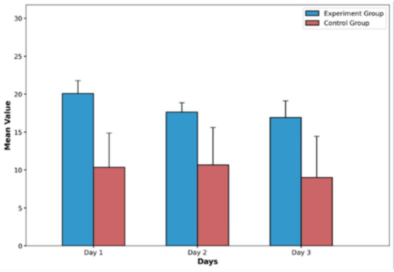

A. grooming > 1s

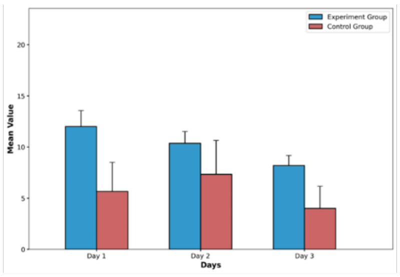

B. grooming > 4s

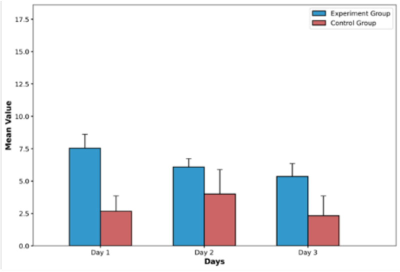

C. grooming > 7s

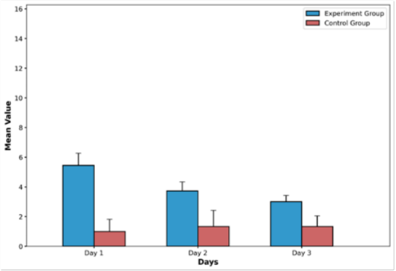

D. grooming > 10s

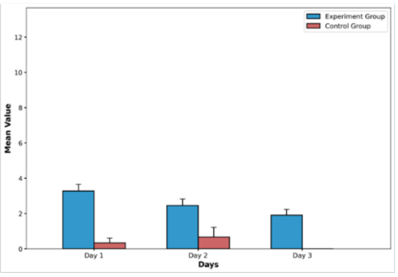

E. grooming > 13s

Fig.S2. Results of grooming behavior under different time durations. A. Results of behavior lasting > 1s. B. Results of behavior lasting > 4s. C. Results of behavior lasting > 7s. D. Results of behavior lasting > 10s. E. Results of behavior lasting > 13s.

Fig.S2 illustrates the application of time threshold filtering for grooming behavior using thresholds of 1 second, 4 seconds, 7 seconds, 10 seconds, and 13 seconds. If a threshold of only 1 second or 4 seconds is used for statistical purposes, the occurrence of grooming in the treatment group shows a difference only on the first day compared to the control group.

As illustrated in Figure S2, as the time threshold for statistical counting increases, both groups show a decline in grooming occurrences; however, the control group exhibits noticeably fewer prolonged grooming behaviors. At thresholds of 10 seconds and 13 seconds, a discernible difference in grooming counts between the two groups emerges. Consequently, this study adopts a threshold of 13 seconds as the time criterion for grooming behavior. Grooming behavior displayed a clear disparity between the drug-treated and control groups on the first day of withdrawal, with a progressive daily decline.

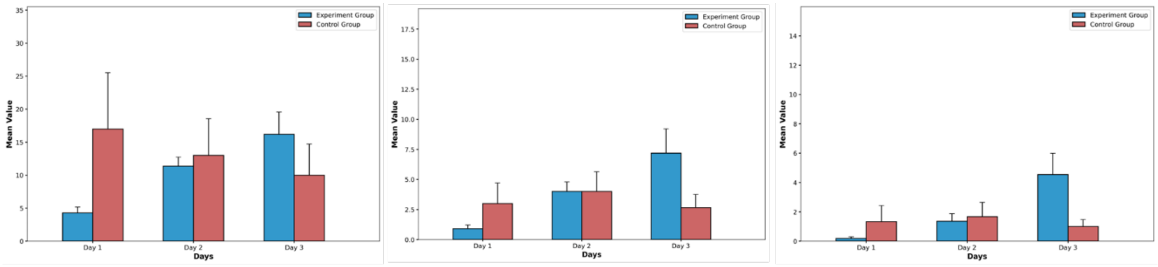

A. head-raising > 1s B. head-raising > 4s C. head-raising > 7s

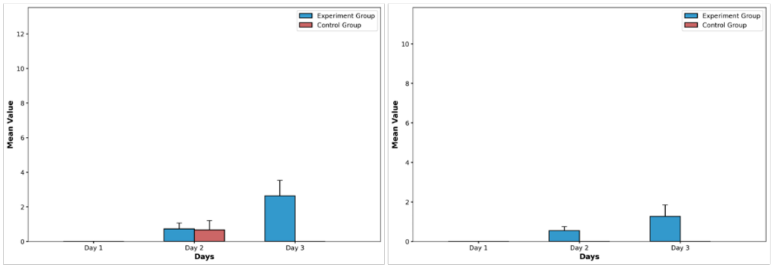

D. head-raising > 10s E. head-raising > 13s

Fig.S3. Results of head-raising behavior under different time durations. A. Results of behavior lasting > 1s. B. Results of behavior lasting > 4s. C. Results of behavior lasting > 7s. D. Results of behavior lasting > 10s. E. Results of behavior lasting > 13s.

As shown in Fig.S3, when the head-raising behavior is greater than 10 seconds or 13 seconds, the behavior appears rarely enough to be used as a basis for judgment. When the behavior is greater than 1 second, 4 seconds, and 7 seconds, it can be found that the experimental group's head-raising behaviors increased every day, while the control group's changes are not obvious. This suggests that head raising may be a milder withdrawal behavior that becomes more apparent as the frequency of other behaviors declines. Further experiments will help clarify its occurrence patterns.

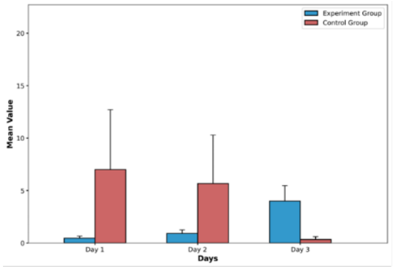

A. rearing ＞1s

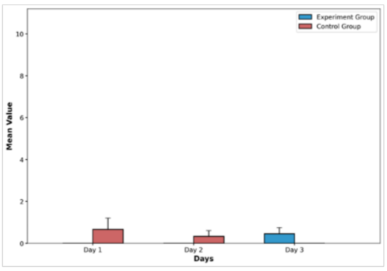

B. rearing ＞4s

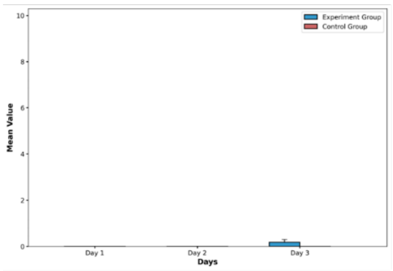

C. rearing ＞7s

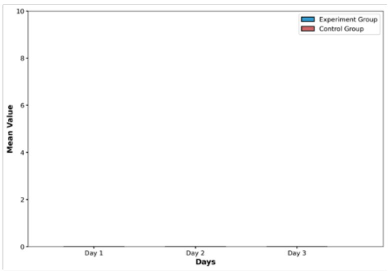

D. rearing ＞10s

Fig.S4. Results of rearing behavior under different time durations. A. Results of behavior lasting > 1s. B. Results of behavior lasting > 4s. C. Results of behavior lasting > 7s. D. Results of behavior lasting > 10s.

As illustrated in Fig.S4, rearing behavior greater than 7 seconds or 10 seconds is rare. In contrast, the differences between the experimental and control groups are more pronounced in rearing behavior greater than 1 second or 4 seconds. Rearing behavior is rare in the experimental group on the first two days and begins to increase on the third day. On the contrary, the control group had more rearing behaviors in the first two days and decreased by the third day.

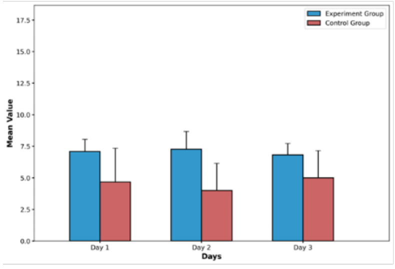

A. genital licking ＞1s

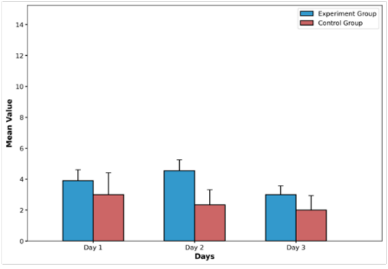

B. genital licking ＞4s

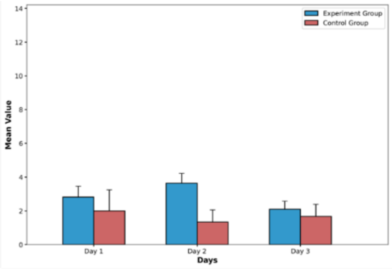

C. genital licking ＞7s

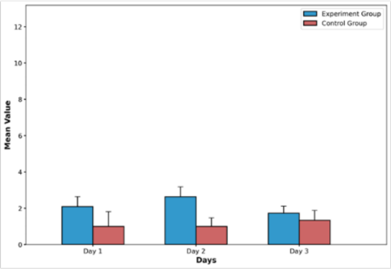

D. genital licking ＞10s

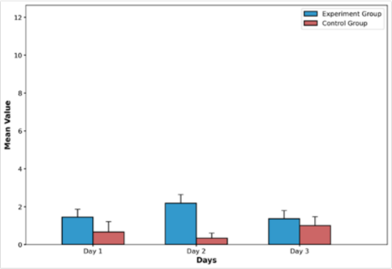

E. genital licking ＞13s

Fig.S5. Results of genital licking behavior under different time durations. A. Results of behavior lasting > 1s. B. Results of behavior lasting > 4s. C. Results of behavior lasting > 7s. D. Results of behavior lasting > 10s. E. Results of behavior lasting >13s.

As shown in Fig.S5, the difference in the frequency of genital licking is not significant over several days at duration thresholds greater than 1 second. As the increase in threshold becomes progressively more pronounced, the frequency of this behavior in the experimental group initially increases on the second day and then decreases on the third day, with a significant difference occurring on the second day.

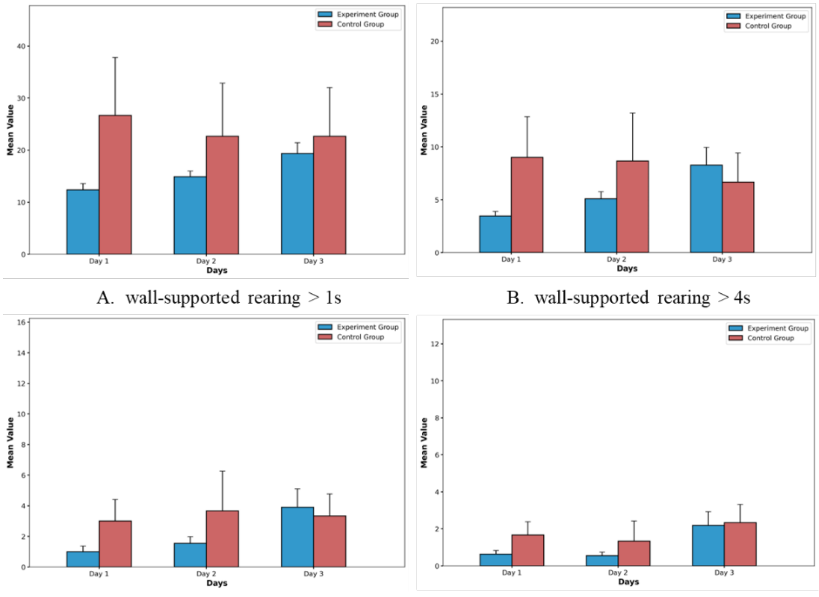

A. wall-supported rearing > 1s

B. wall-supported rearing > 4s

C. wall-supported rearing > 7s

D. wall-supported rearing > 10s

Fig.S6. Results of wall-supported rearing behavior under different time durations. A. Results of behavior lasting > 1s. B. Results of behavior lasting > 4s. C. Results of behavior lasting > 7s. D. Results of behavior lasting > 10s.

As shown in Fig.S6, the wall-supporting rearing behavior gradually decreased as the time threshold increased. There is significant variability in wall-supported rearing behavior in the first two days, with no significant difference by the third day.

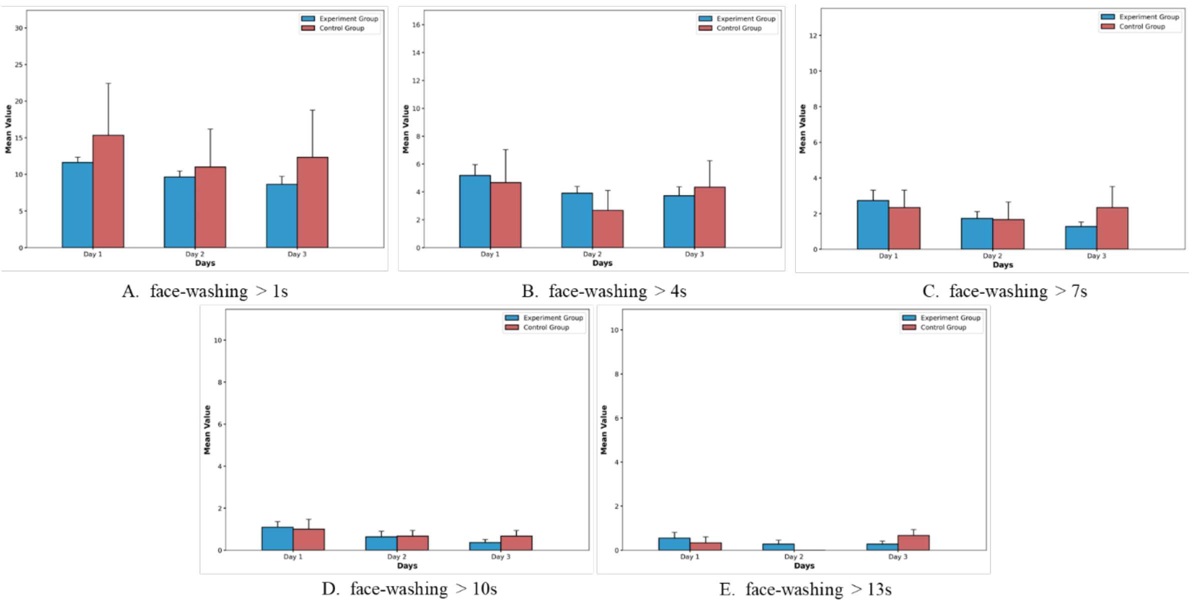

Fig.S7. Results of face-washing behavior under different time durations. A. Results of behavior lasting > 1s. B. Results of behavior lasting > 4s. C. Results of behavior lasting > 7s. D. Results of behavior lasting > 10s. E. Results of behavior lasting > 13s.

Fig.S7 displays the statistical results for face-washing behavior under different time thresholds. Despite increasing the screening duration, the differences in face-washing behavior between the drug-treated and control groups remained insignificant.

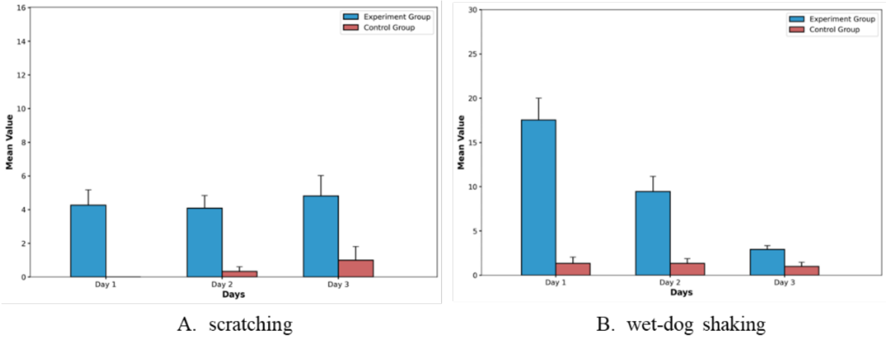

Fig.S8. A. Results of scratching behavior. B. Results of wet-dog shaking behavior.

Fig.S8-A illustrates the results of the identification of scratching behaviors, with significant differences in both groups over the three days.

Fig.S8-B illustrates the recognition results for the wet-dog shaking behavior. This behavioral indicator exhibited significant intergroup differences. From the first day of withdrawal, the number of shakes in the drug-treated group rapidly decreased, aligning with the control group by the third day.

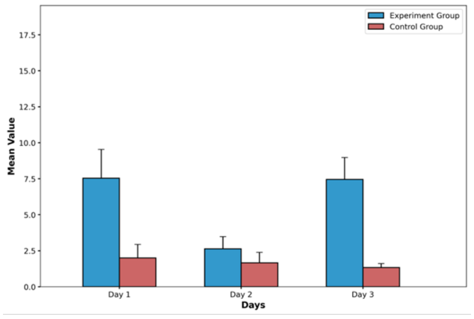

teeth chattering

Fig.S9. Results of teeth chattering behavior.

Fig. S9 presents the automatic recognition results for teeth chattering. Teeth chattering was more frequent in the drug-treated group on the first and third days, though it did not exhibit clear temporal regularity.
